# Traumatic brain injury, changes in plasma amyloid, tau, and neurodegenerative biomarkers, and dementia risk

**DOI:** 10.1002/alz.70611

**Published:** 2025-09-15

**Authors:** Alexa E. Walter, James R. Pike, Josef Coresh, Ramon Diaz‐Arrastia, David Menon, Rebecca F. Gottesman, Priya Palta, Andrea L. C. Schneider

**Affiliations:** ^1^ Department of Neurology University of Pennsylvania Perelman School of Medicine Philadelphia Pennsylvania USA; ^2^ Optimal Aging Institute New York University Grossman School of Medicine New York New York USA; ^3^ Department of Anaesthesia University of Cambridge Cambridge UK; ^4^ National Institute of Neurological Disorders and Stroke Intramural Research Program Bethesda Maryland USA; ^5^ Department of Neurology University of North Carolina at Chapel Hill Chapel Hill North Carolina USA; ^6^ Department of Biostatistics, Epidemiology, and Informatics University of Pennsylvania Perelman School of Medicine Philadelphia Pennsylvania USA

**Keywords:** Cohort study, dementia, plasma biomarkers, TBI, traumatic brain injury

## Abstract

**INTRODUCTION:**

Long‐term trajectories of plasma biomarkers in relation to incident traumatic brain injury (TBI) and whether TBI modifies associations of biomarkers with dementia risk are unknown.

**METHODS:**

One thousand fifty Atherosclerosis Risk in Communities (ARIC) study participants without prior TBI had amyloid beta (Aβ_42_/Aβ_40_), phosphorylated‐tau181 (pTau181), neurofilament light (NfL), and glial fibrillary acidic protein (GFAP) measured from plasma collected in 1993 to 1995, 2011 to 2013, and 2016 to 2019. Linear mixed‐effects models estimated biomarker trajectories associated with TBI and Cox proportional hazards models determined if TBI modified associations of biomarkers with incident dementia through December 31, 2020.

**RESULTS:**

After the median time of incident TBI, Aβ_42_/Aβ_40_ levels remained lower for 9.3 years, and pTau181, NfL, and GFAP remained elevated for 8.5, >13.8, and 12.7 years, respectively. There was evidence of additive interaction by TBI in associations of log_2_NfL with incident dementia (*p* = 0.024).

**DISCUSSION:**

TBI alters trajectories of plasma biomarkers of neurodegeneration for approximately a decade after the injury and modifies associations of NfL with dementia risk.

**Highlights:**

- Our findings provide evidence that TBI fundamentally alters trajectories of plasma biomarkers of AD‐related pathology, neuronal degeneration, and astrogliosis for approximately a decade after the injury.
- Further, our findings also suggest that an incident TBI event adds to and interacts with ongoing neurodegenerative processes to increase the risk of later life dementia.
- These results suggest that the pathologic processes underlying post‐TBI dementia are heterogeneous, that individuals with preclinical changes in neurodegenerative biomarkers may be more susceptible to TBI (i.e., that associations are bidirectional), or a combination thereof.

## 1 BACKGROUND

Blood‐based biomarkers of Alzheimer's disease (AD)‐related pathology, neuronal injury, and astrogliosis show promise as clinical screening tools for dementia.^1^ These same biomarkers are also of interest in traumatic brain injury (TBI), which is recognized as a modifiable risk factor for dementia,^2^ and where glial fibrillary acidic protein (GFAP), in combination with ubiquitin carboxyl‐terminal hydrolase L1 (UCHL1), is cleared for clinical use in the acute injury setting. Given the implications for clinical use of these plasma biomarkers in both dementia and TBI, there is a need to better understand trajectories from midlife to late life and from before TBI to after TBI and to understand how TBI may influence the associations of these biomarkers with dementia risk.

Prior studies suggested that neurofilament light (NfL) and GFAP remained elevated for at least 5 years after TBI compared to individuals without TBI,^3^, ^4^ whereas studies investigating trajectories of AD‐related biomarkers (amyloid beta [Aβ] and tau protein isoforms) have generally had shorter‐term follow‐up and more heterogeneous results.^3^, ^5^, ^6^ The trajectories of these plasma biomarkers in the decade(s) following injury remain unclear. Further, it is unknown how TBI may influence associations of blood‐based biomarkers with later dementia risk. Greater understanding of the interplay between TBI and biomarkers over the life course in relation to later‐life dementia risk has the potential to provide mechanistic insights that may inform targets for future dementia prevention efforts among individuals with TBI.

Leveraging data collected over 26 years (late midlife to late life) from community‐dwelling participants in the Atherosclerosis Risk in Communities (ARIC) study, we sought to characterize temporal changes in plasma biomarkers of neurodegeneration in relation to incident TBI and to evaluate whether incident TBI status modifies the associations of neurodegenerative biomarkers with incident dementia risk.

## 2 METHODS

### 2.1 Study population and design

**FIGURE 1:**
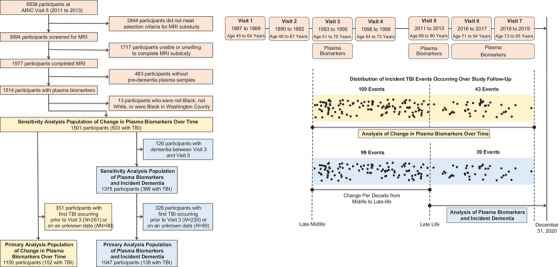
Study design and flowchart of participants selected for analyses. ARIC, Atherosclerosis Risk in Communities; MRI, magnetic resonance imaging; TBI, traumatic brain injury.

The ARIC study is an ongoing community‐based cohort of 15,792 largely Black and White participants from four US communities (Forsyth County, North Carolina; Jackson, Mississippi; Washington County, Maryland; and selected suburbs of Minneapolis, Minnesota).^7^ Participants from the North Carolina, Maryland, and Minnesota sites are representative of the underlying population, while only Black participants were recruited at the Mississippi site. Participants were enrolled in the period 1987 to 1989 and attended subsequent in‐person visits in 1990 to 1992 (Visit 2), 1993 to 1995 (Visit 3), 1996 to 1998 (Visit 4), 2011 to 2013 (Visit 5), 2016 to 2017 (Visit 6), and 2018 to 2019 (Visit 7), with follow‐up ongoing (Figure 1). Participants or their proxies completed annual (1987 to 2011) and semi‐annual (starting 2012) phone‐based assessments and granted access to hospitalization records and death certificates. Records from the Centers for Medicare & Medicaid Services (CMS) were obtained for participants aged 65+ years who were enrolled in fee‐for‐service part B (1991 to 2018).

Details of the design and selection of the subsample of ARIC participants with plasma biomarker data were described previously.^8^ This sample is based on participants who previously enrolled in a brain magnetic resonance imaging (MRI) substudy at ARIC Visit 5 (2011 to 2013).^9^, ^10^ Of the 1977 participants in the Visit 5 substudy, 463 participants did not have pre‐dementia plasma samples (exclusion of these individuals creates a population without dementia at the time of the first biomarker measurement at Visit 3, 1993 to 1995). Thirteen participants of non‐Black race, non‐White race, or of Black race in Maryland were subsequently excluded due to small sample sizes and race/center aliasing.^7^ From this remaining population, a total of 1501 participants had plasma biomarkers measured during at least two of three possible time points (Visit 3, 1993 to 1995; Visit 5, 2011 to 2013; and Visits 6/7, 2016 to 2019) (eFigure‐1).

In our primary analyses, the relationship of incident TBI with changes in plasma biomarkers over time was examined in 1150 participants after excluding 351 participants with incident TBI occurring prior to Visit 3 (1993 to 1995) or on an unknown date. An evaluation of whether TBI status modifies the associations of plasma biomarkers with incident dementia occurring after Visit 5 (2011 to 2013) was performed among 1047 participants without a diagnosis of dementia occurring between Visit 3 (1993 to 1995) and Visit 5 (2011 to 2013, *n* = 126 excluded) and with first TBI occurring after Visit 3 (1993 to 1995) (*n* = 328 excluded). Characteristics of included versus excluded participants are shown for each analysis in eTables 1 and 2. In sensitivity analyses, we included individuals with TBI occurring prior to Visit 3 or on an unknown date to better examine lifetime history of TBI, resulting in 1501 participants included in analyses evaluating changes in plasma biomarkers over time and 1375 participants included in incident dementia analyses.

The ARIC study was approved by Institutional Review Boards at all participating institutions and written informed consent was obtained from each participant or their legally authorized representative at each study visit. The ARIC study is performed in accordance with the ethical standards of the Declaration of Helsinki.

**RESEARCH IN CONTEXT**

1. **Systematic review**: The authors reviewed the literature using PubMed. Prior studies investigated TBI with short‐term trajectories of biomarkers after TBI and associations of TBI with dementia risk. These relevant publications are appropriately cited.
2. **Interpretation**: Our findings provide evidence that TBI fundamentally alters trajectories of plasma biomarkers of AD‐related pathology, neuronal degeneration, and astrogliosis for approximately a decade after the injury. Further, our findings also suggest that an incident TBI event adds to and interacts with ongoing neurodegenerative processes to increase the risk of later‐life dementia.
3. **Future directions**: This manuscript provides evidence suggesting that the pathologic processes underlying post‐TBI dementia are heterogeneous, that individuals with preclinical changes in neurodegenerative biomarkers may be more susceptible to TBI (i.e., that associations are bi‐directional), or a combination thereof – future studies are needed to further elucidate mechanisms underlying associations between TBI and dementia.

### 2.2 Traumatic brain injury

Time‐varying TBI and dates of TBI events are defined using three sources of data (eTable 3), as previously described^11^, ^12^: (1) self‐reported lifetime history data collected from questionnaires administered at in‐person visits, (2) continuously collected *International Classification of Diseases Ninth and Tenth Revisions* (ICD‐9/10) codes from ARIC study hospitalization data, and (3) continuously collected outpatient/hospital‐based ICD‐9/10 codes available via linkage to CMS data. Self‐reported data collected at in‐person visits include information on the number and year(s) of head injury(ies) that required medical care or were associated with loss of consciousness. The random point method was used to impute the month and date of self‐reported TBIs.^13^ ICD‐9/10 codes used to identify TBI cases are consistent with the Centers for Disease Control and Prevention surveillance definition for TBI.^14^, ^15^ In secondary analyses, we considered the number of TBIs (0, 1, 2+), the severity of the first TBI (no TBI, mild TBI, moderate/severe/penetrating TBI; defined for injuries identified by ICD codes in accordance with the Department of Defense ICD‐9/10 code‐based criteria^16^), and the timing of the incident TBI event (before or after Visit 5, 2011 to 2013). In sensitivity analyses, we considered self‐reported and ICD‐9/10 code‐defined TBIs separately.

### 2.3 Plasma biomarkers

Plasma samples from ARIC Visit 3 (1993–1995), Visit 5 (2011–2013), and Visits 6/7 (2016–2019) were analyzed using the Neurology 4‐Plex E assay on the Quanterix (Billerica, MA) Simoa platform using a HD‐X instrument (eMethods, eTable 4, eTable 5). Aβ_40_, Aβ_42_, NfL, and GFAP were measured using a multiplex assay, and pTau181 was measured using a singleplex assay. Consistent with prior analyses,^8^ the Aβ_42_/Aβ_40_ ratio was calculated and inverted, and pTau181, NfL, and GFAP were base 2 log‐transformed to meet statistical modeling assumptions of normality. In sensitivity analyses, biomarker measurements occurring within 1 year after a TBI event were excluded (*n* = 5 observations at Visit 5 [2011 to 2013] and *n* = 3 observations at Visit 6/7 [2016 to 2019]).

### 2.4 Dementia

All‐cause dementia and dates of dementia diagnosis in ARIC are defined according to a three‐level dementia ascertainment protocol^17^ (eTable 6) that is based on the National Institute on Aging‐Alzheimer's Association criteria^18^, ^19^ and the *Diagnostic and Statistical Manual of Mental Disorders*, Fifth Edition.^20^ Dementia is defined from in‐person cognitive evaluations and informant interviews starting at ARIC Visit 5 (2011 to 2013), data collected from telephone calls with participants and/or informants, and continuously collected hospitalization ICD‐9/10 code and death certificate code data.

### 2.5 Statistical analysis

Statistical analyses were conducted using SAS version 9.4 (SAS Institute, Cary, NC, USA) and Stata 14.0 (StataCorp, College Station, TX, USA). Statistical significance was defined as a two‐sided *p* < 0.05.

We estimated associations of time‐varying TBI with change per decade in units of SDs of inverted Aβ_42_/Aβ_40_ ratio, log_2_pTau181, log_2_NfL, and log_2_GFAP by fitting covariate adjusted two‐level linear mixed effects models (eMethods) that specified time from Visit 3 (1993–1995) as the timescale, included a random intercept and random time slope, and employed an unstructured covariance matrix. Covariates included age, sex, race/center, and education as time‐invariant covariates and estimated glomerular filtration rate and body mass index as time‐varying covariates (eMethods). An interaction was specified between each time‐invariant covariate and time. We used multiple imputation by chained equations to impute missing covariates and inverse probability weighting to account for selection bias and informative attrition (eMethods). Model coefficients of interest included: (1) ‘time since baseline,’ which estimated the change per decade in biomarker levels for individuals with no TBI/prior to TBI, (2) ‘time since baseline+interaction of TBI*time since baseline,’ which estimated the change per decade in biomarker levels for individuals after incident TBI, (3) ‘interaction of TBI*time since baseline,’ which estimated the difference in slope per decade in biomarker levels after incident TBI compared to no TBI/prior to TBI, and (4) ‘TBI+TBI*time since baseline,’ which estimated the difference in biomarker levels at the median time (12.8 years post‐Visit 3, 1993–1995) of incident TBI compared to no TBI/prior to TBI. We formally tested for effect modification by age, sex, race, education, apolipoprotein E ε4 genotype (0 alleles; 1 or 2 alleles; measured using the TaqMan assay [Applied Biosystems, Foster City, CA]), and cognitive status (normal; mild cognitive impairment or dementia).

We investigated if time‐varying TBI status modifies associations of late midlife (Visit 3, 1993–1995), late‐life (Visit 5, 2011–2013), and change from late midlife to late‐life in inverted Aβ_42_/Aβ_40_ ratio, log_2_pTau181, log_2_NfL, and log_2_GFAP, with incident dementia occurring after Visit 5 (2011–2013) using cause‐specific Cox proportional hazards regression models that employed the Efron method to handle tied onset times. Model assumptions were confirmed by evaluating Schoenfeld and Martingale residuals. Follow‐up time extended from Visit 5 (2011–2013) to incident dementia, loss to follow‐up/study withdrawal, death, or administrative censoring on 12/31/2020. Covariate adjusted Cox models included sampling weights to account for selection bias and used multiple imputation by chained equations to impute missing covariates (eMethods). We evaluated for effect modification by TBI status in associations of each biomarker with incident dementia on the (1) multiplicative scale (i.e., combined effect is larger or smaller than the product of individual effects) by including the product of each biomarker with TBI in the statistical model and (2) additive scale (i.e., combined effect is larger or smaller than the sum of individual effects) using the exponentiated relative excess risk due to interaction (RERI).^21^ Interaction values equal to 1 indicate no evidence of interaction, values > 1 indicate positive interaction, and values < 1 indicate negative interaction.

## 3 RESULTS

### 3.1 Change in plasma biomarkers

In the primary analysis population, for change in plasma biomarkers (*N* = 1150), the mean (standard deviation [SD]) age in midlife (Visit 3, 1993 to 1995) was 59.1 (5.2) years, 65.8% were female, 28.7% were Black, and 13.2% had an incident TBI occur at a median of 12.8 years after Visit 3 (interquartile range [IQR] = 5.3 to 18.3) (median age at first TBI = 72.5 years, IQR = 66.1 to 79.3) (Table 1). Participants with an incident TBI versus without were slightly older (60.2 vs 58.9 years) and more likely to be diagnosed with dementia during study follow‐up (34.9%, median age at diagnosis 85.8 years [IQR = 81.2 to 88.8] vs 24.7%, median age at diagnosis 83.4 years [IQR = 78.5 to 87.6]).

**TABLE 1:** Characteristics of primary analysis population of change in plasma biomarkers over time stratified by incident traumatic brain injury (TBI) status (*N* = 1150).

	**Overall**	**No TBI (*N* = 998)**	**TBI (*N* = 152)**
**Visit 3 (1993 to 1995)**			
Age, years	59.1 (5.2) [1150]	58.9 (5.2) [998]	60.2 (5.2) [152]
Female sex	757/1150 (66%)	648/998 (65%)	109/152 (72%)
Race and center			
Black, Jackson, Mississippi	309/1150 (27%)	285/998 (29%)	24/152 (16%)
White, Washington County, Maryland	305/1150 (27%)	255/998 (26%)	50/152 (33%)
White, Minneapolis suburbs, Minnesota	267/1150 (23%)	223/998 (22%)	44/152 (29%)
White, Forsyth County, North Carolina	247/1150 (22%)	216/998 (22%)	31/152 (20%)
Black, Forsyth County, North Carolina	22/1150 (2%)	19/998 (2%)	3/152 (2%)
Education			
Less than completed high school	154/1150 (13%)	137/998 (14%)	17/152 (11%)
High school, GED, or vocational school	481/1150 (42%)	416/998 (42%)	65/152 (43%)
Some college, graduate, or professional school	515/1150 (45%)	445/998 (45%)	70/152 (46%)
One or more apolipoprotein E alleles	304/1088 (28%)	264/945 (28%)	40/143 (28%)
Cigarette use			
Current	129/1149 (11%)	114/997 (11%)	15/152 (10%)
Former	436/1149 (38%)	374/997 (38%)	62/152 (41%)
Never	584/1149 (51%)	509/997 (51%)	75/152 (49%)
Alcohol use			
Current	595/1148 (52%)	514/997 (52%)	81/151 (54%)
Former	237/1148 (21%)	205/997 (21%)	32/151 (21%)
Never	316/1148 (28%)	278/997 (28%)	38/151 (25%)
Diabetes	94/1149 (8%)	84/997 (8%)	10/152 (7%)
Hypertension	422/1150 (37%)	371/998 (37%)	51/152 (34%)
Body mass index, kg/m^2^	28.0 (5.1) [1150]	27.9 (5.0) [998]	28.4 (5.6) [152]
Estimated glomerular filtration rate, mL/min/1.73 m^2^	91.5 (14.0) [1102]	91.7 (13.8) [954]	90.1 (15.4) [148]
TBI frequency			
No TBI	998/1150 (87%)	998/998 (100%)	0/152 (0%)
1 TBI	78/1150 (7%)	0/998 (0%)	78/152 (51%)
2+ TBIs	74/1150 (6%)	0/998 (0%)	74/152 (49%)
TBI severity			
No TBI	998/1131 (88%)	998/998 (100%)	0/133 (0%)
Mild TBI	99/1131 (9%)	0/998 (0%)	99/133 (74%)
Moderate, severe, penetrating TBI	34/1131 (3%)	0/998 (0%)	34/133 (26%)
**Plasma Assays**			
Amyloid beta (Aβ) 42, pg/mL			
Visit 3 (1993 to 1995)	3.77 (1.77) [1150]	3.80 (1.78) [998]	3.59 (1.68) [152]
Visit 5 (2011 to 2013)	6.35 (1.89) [1149]	6.32 (1.89) [997]	6.56 (1.85) [152]
Visits 6 and 7 (2016 to 2019)	6.68 (1.88) [379]	6.65 (1.87) [343]	7.02 (1.95) [36]
Amyloid beta 40, pg/mL			
Visit 3 (1993 to 1995)	59.2 (28.7) [1150]	59.4 (28.5) [998]	58.0 (29.6) [152]
Visit 5 (2011 to 2013)	108.8 (25.4) [1149]	107.8 (25.4) [997]	115.1 (24.5) [152]
Visits 6 and 7 (2016 to 2019)	112.4 (24.0) [379]	112.1 (24.0) [343]	114.9 (23.5) [36]
Aß42/Aß40 ratio			
Visit 3 (1993 to 1995)	0.073 (0.046) [1150]	0.073 (0.046) [998]	0.073 (0.045) [152]
Visit 5 (2011 to 2013)	0.059 (0.014) [1149]	0.059 (0.014) [997]	0.058 (0.013) [152]
Visits 6 and 7 (2016 to 2019)	0.060 (0.012) [379]	0.060 (0.013) [343]	0.061 (0.009) [36]
Phosphorylated tau‐181			
Visit 3 (1993 to 1995)	1.57 (0.96) [1149]	1.56 (0.94) [997]	1.65 (1.09) [152]
Visit 5 (2011 to 2013)	3.22 (1.85) [1147]	3.20 (1.83) [995]	3.35 (1.96) [152]
Visits 6 and 7 (2016 to 2019)	3.33 (1.67) [379]	3.31 (1.65) [343]	3.51 (1.93) [36]
Log2 phosphorylated tau‐181			
Visit 3 (1993 to 1995)	0.44 (0.80) [1149]	0.44 (0.78) [997]	0.45 (0.93) [152]
Visit 5 (2011 to 2013)	1.51 (0.68) [1147]	1.50 (0.68) [995]	1.57 (0.70) [152]
Visits 6 and 7 (2016 to 2019)	1.58 (0.66) [379]	1.58 (0.65) [343]	1.62 (0.75) [36]
Neurofilament light chain, pg/mL			
Visit 3 (1993 to 1995)	12.9 (8.1) [1150]	12.7 (8.3) [998]	14.2 (6.4) [152]
Visit 5 (2011 to 2013)	25.6 (13.8) [1149]	25.1 (13.7) [997]	28.6 (14.4) [152]
Visits 6 and 7 (2016 to 2019)	29.5 (15.9) [379]	29.1 (15.7) [343]	33.6 (17.4) [36]
Log2 neurofilament light chain			
Visit 3 (1993 to 1995)	3.51 (0.69) [1150]	3.49 (0.69) [998]	3.68 (0.67) [152]
Visit 5 (2011 to 2013)	4.50 (0.71) [1149]	4.47 (0.71) [997]	4.68 (0.66) [152]
Visits 6 and 7 (2016 to 2019)	4.72 (0.66) [379]	4.70 (0.66) [343]	4.91 (0.66) [36]
Glial fibrillary acidic protein, pg/mL			
Visit 3 (1993 to 1995)	108.5 (67.3) [1150]	107.8 (69.6) [998]	112.9 (49.4) [152]
Visit 5 (2011 to 2013)	199.6 (103.3) [1149]	195.9 (101.6) [997]	223.5 (111.1) [152]
Visits 6 and 7 (2016 to 2019)	212.3 (102.1) [379]	210.4 (99.0) [343]	230.5 (128.2) [36]
Log2 glial fibrillary acidic protein			
Visit 3 (1993 to 1995)	6.59 (0.69) [1150]	6.58 (0.69) [998]	6.68 (0.65) [152]
Visit 5 (2011 to 2013)	7.47 (0.69) [1149]	7.45 (0.69) [997]	7.65 (0.67) [152]
Visits 6 and 7 (2016 to 2019)	7.59 (0.64) [379]	7.57 (0.64) [343]	7.69 (0.65) [36]
Dementia on or before 2020	300/1150 (26%)	247/998 (25%)	53/152 (35%)
Death on or before 2020	313/1150 (27%)	262/998 (26%)	51/152 (34%)

*Note*: Data are *n* (%) or mean (SD) [total]. Denominators for percentages are based on the number of participants with complete data.

Abbreviations: GED, General Educational Development credential; pg, picograms; SD, standard deviations.

**FIGURE 2:**
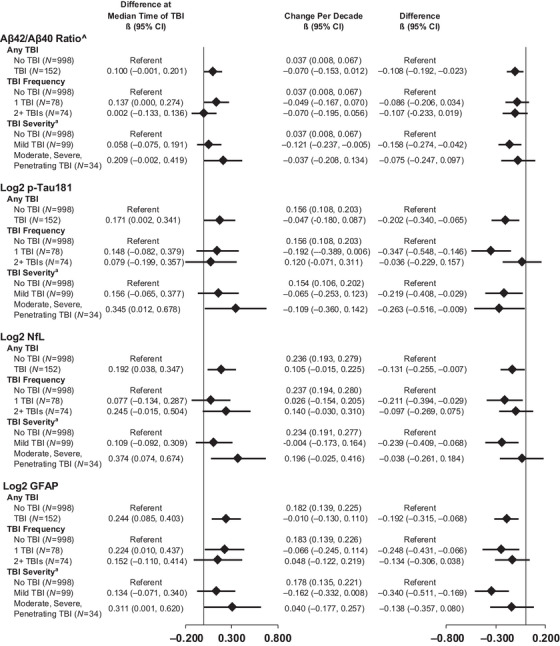
Covariate‐adjusted change (95% CI) in standardized plasma biomarkers by traumatic brain injury (TBI) status, the ARIC study 1993 to 2019 (*N* = 1150). Aβ, amyloid beta; CI, confidence interval; GFAP, glial fibrillary acidic protein; NfL, neurofilament light chain; pTau181, phosphorylated tau‐181; TBI, traumatic brain injury. ^Indicates that the Aβ42/Aβ40 ratio was inverted so that higher values denote greater risk. ^a^TBI severity data were only available in a subset of participants (*N* = 1131). The analytic sample was restricted to pre‐dementia plasma biomarkers measured using the Quanterix Simoa platform. Parameter estimates generated from linear mixed‐effects models. First TBI defined as occurring 12.8 years after Visit 3, and second TBI defined as occurring 19.0 years after Visit 3 (median injury times). Biomarkers standardized to Visit 3 by mean‐centering and dividing by 0.0459 for Aβ42/Aβ40 ratio, 0.8033 for log2 pTau181, 0.6920 for log2 NfL, and 0.6867 for log2 GFAP. Models adjusted for age, sex, race‐center, and education as time‐invariant covariates and estimated glomerular filtration rate and body mass index as time‐varying covariates. An interaction was specified between each time‐invariant covariate and time. Multiple imputation by chained equations was employed to impute missing covariates. Inverse probability weighting was used to account for selection bias and informative attrition.

Figure 2 shows the covariate‐adjusted change in standardized plasma biomarkers by TBI incidence, frequency, and severity. At the median time of TBI (12.8 years after Visit 3, 1993 to 1995), levels of the inverted Aβ_42_/Aβ_40_ ratio, log_2_pTau181, log_2_NfL, and log_2_GFAP were higher among individuals with incident TBI versus without, with the strongest association observed for log_2_GFAP (0.244 [95% CI = 0.085 to 0.403] SD). There was no evidence for effect modification by age, sex, race, education, APOE genotype, or cognitive status (all *p* > 0.1) (eTable 7).

**FIGURE 3:**
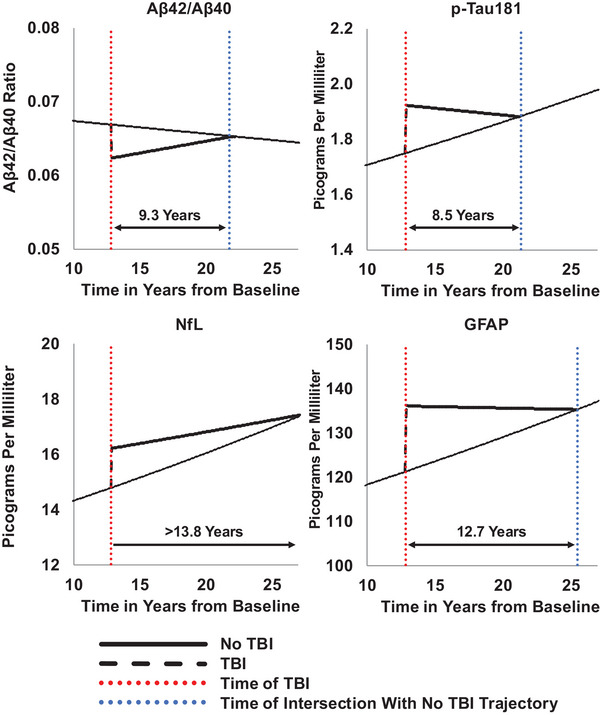
Covariate‐adjusted, model‐based estimates of change in plasma biomarkers over time by traumatic brain injury (TBI) status, the ARIC study 1993 to 2019 (*N* = 1150). Aβ, amyloid beta; CI, confidence interval; GFAP, glial fibrillary acidic protein; NfL, neurofilament light chain; pTau181, phosphorylated tau‐181; TBI, traumatic brain injury. The analytic sample was restricted to pre‐dementia plasma biomarkers measured using the Quanterix Simoa platform. TBI defined as occurring 12.8 years after Visit 3 (median injury time). Parameter estimates generated from linear mixed‐effects models that adjusted for age, sex, race‐center, and education as time‐invariant covariates and estimated glomerular filtration rate and body mass index as time‐varying covariates. An interaction was specified between each time‐invariant covariate and time. Multiple imputation by chained equations was employed to impute missing covariates. Inverse probability weighting was used to account for selection bias and informative attrition. pTau181, NfL, and GFAP were base 2 log‐transformed prior to analysis and then converted back to their original scale for the visualization.

After the median time of incident TBI (12.8 years after Visit 3, 1993 to 1995), compared to individuals without TBI, levels remained lower for 9.3 years for the Aβ_42_/Aβ_40_ ratio and remained higher for 8.5 years for pTau181, over 13.8 years for NfL, and 12.7 years for GFAP. Figure 3 depicts these estimated trajectories on the clinically relevant (non‐log_2_‐transformed) scale.

In secondary analyses by TBI frequency and severity, biomarker levels tended to return to non‐/pre‐injury levels more slowly (i.e., smaller change per decade) after two or more injuries versus after one injury (log_2_pTau181, log_2_NFL, and log_2_GFAP) and after more versus less severe injuries (log_2_NFL and log_2_GFAP) (Figure 2, eFigure 2, eFigure 3). In sensitivity analyses considering self‐reported and ICD‐9/10 code defined TBIs separately, the change per decade for all biomarkers was attenuated for self‐reported TBI and was of similar magnitude for ICD‐9/10 code‐defined TBI compared to the primary analyses (eTable 8, eFigure 4, eFigure 5). In sensitivity analyses excluding biomarker observations in which a participant had TBI within 1 year before a visit, results were similar to the main findings (eFigure 6).

In the sensitivity analysis population including individuals with TBI prior to the first biomarker measurement at Visit 3 (1993 to 1995) (*N* = 1501, eTable 9), results for the covariate‐adjusted changes in standardized plasma biomarkers by TBI status were attenuated compared to the primary analysis population (eTable 10).

### 3.2 Plasma biomarkers and incident dementia

Among the 1047 included in the primary analysis population used to evaluate whether TBI status modified the association between plasma[Fig alz70611-fig-0004] biomarkers and incident dementia occurring after Visit 5 (2011 to 2013), the mean (SD) age was 76.6 (5.3) years at Visit 5 (2011 to 2013), 65.9% were female, 28.9% were Black, and 13.2% had an incident TBI (eTable 11).

**FIGURE 4 alz70611-fig-0004:**
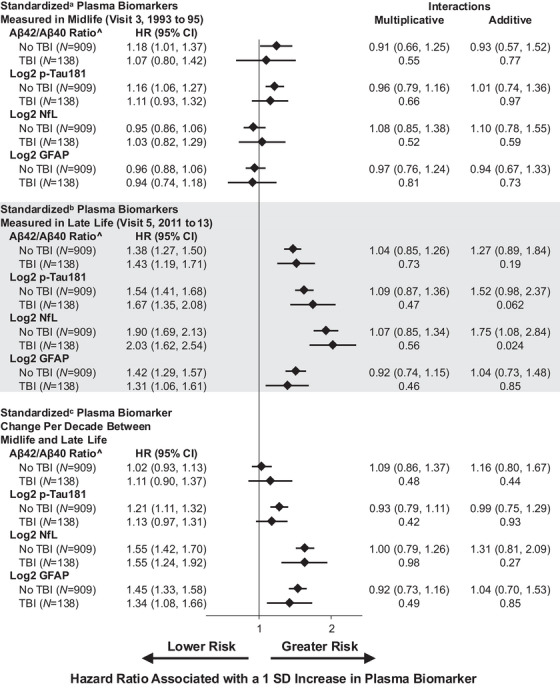
Covariate‐adjusted association of standardized plasma biomarkers with incident dementia in late‐life by any traumatic brain injury (TBI) status, the ARIC Study 2011 to 2020 (*N* = 1047). Aβ, amyloid beta; CI, confidence interval; GFAP, glial fibrillary acidic protein; HR, hazard ratio; NfL, neurofilament light chain; pTau181, phosphorylated tau‐181; TBI, traumatic brain injury. ^Aβ42/Aβ40 ratio inverted so that higher values denote greater risk of incident dementia. ^a^Biomarkers standardized to Visit 3 by mean‐centering and dividing by 0.0459 for Aβ42/Aβ40 ratio, 0.7997 for log2 pTau181, 0.6824 for log2 NfL, and 0.6885 for log2 GFAP. ^b^Biomarkers standardized to Visit 5 by mean‐centering and dividing by 0.0143 for Aβ42/Aβ40 ratio, 0.6631 for log2 pTau181, 0.6990 for log2 NfL, and 0.6951 for log2 GFAP. ^c^Change in biomarkers per decade standardized by mean‐centering and dividing by 0.0270 for Aβ42/Aβ40 ratio, 0.4506 for log2 pTau181, 0.3589 for log2 NfL, and 0.3172 for log2 GFAP. Multiplicative interactions show the HR (95% CI) and *p* value for TBI × biomarker interaction term. Additive interactions show the exponentiated relative excess risk due to interaction (RERI) (95% CI) and *p* values. The analytic sample was restricted to pre‐dementia plasma biomarkers measured using the Quanterix Simoa platform. Hazard ratios and 95% confidence intervals for incident dementia were calculated from cause‐specific Cox proportional hazards regression models. Models adjusted for Visit 5 measures of age, sex, race‐center, education, estimated glomerular filtration rate, and body mass index as time‐invariant covariates. Multiple imputation by chained equations was employed to impute missing covariates. Inverse probability weighting was used to account for selection bias.

Hazard ratios for the associations of each plasma biomarker from Visit 3 (1993 to 1995), Visit 5 (2011 to 2013), and the change per decade from Visit 3 to Visit 5 with incident dementia by time‐varying TBI status are shown in Figure 4. There was evidence for non‐multiplicative, positive additive interaction by TBI in associations of late‐life (Visit 5, 2011 to 2013) log_2_NfL with incident dementia (exponentiated RERI = 1.75, 95% CI = 1.08, 2.84, *p* = 0.024), which indicates that dementia risk in the TBI group is higher overall compared to the no‐TBI groups (e.g., participants at the mean level of NfL in the TBI group have a higher risk of dementia than participants at the mean level of NfL in the no‐TBI group, but the ratio of dementia risk by TBI status is similar across different levels of NfL). There was no evidence of effect modification by incident TBI in associations of any midlife or change from midlife to late‐life biomarkers with dementia risk.

In secondary analyses examining TBI frequency, severity, and timing (eTable 12), there was evidence for interaction by TBI frequency on the multiplicative and additive scales in associations of midlife inverted Aβ_42_/Aβ_40_ ratio (higher in 2+ vs 1 TBI group) and late‐life log_2_pTau181 and log_2_NfL (both higher in 1 vs 2+ TBI group) with incident dementia. There was evidence for multiplicative and additive interaction by TBI severity in associations of midlife inverted Aβ_42_/Aβ_40_ ratio and of the change per decade in log_2_GFAP (both lower among mild vs moderate/severe/penetrating injury) with dementia risk. By timing of TBI, there was effect modification on the multiplicative and additive scales in associations of midlife log_2_GFAP (smaller effect sizes with TBI occurring after vs before Visit 5, 2011 to 2013) and of late‐life log_2_NfL and the change per decade in log_2_NfL and log_2_GFAP (both higher with TBI occurring after vs before Visit 5, 2011 to 2013) with dementia risk. In sensitivity analyses evaluating self‐reported versus ICD‐9/10 code‐defined TBI, similar interaction patterns were observed (eTable 13). In sensitivity analyses excluding biomarker observations in which a participant had TBI within 1 year before a visit, results were similar to the main analyses (eFigure 7).

In the sensitivity analysis population including individuals with TBI prior to the first biomarker measurement at Visit 3 (1993 to 1995) (*N* = 1375, eTable 14), we observed evidence for non‐multiplicative, positive additive interaction by incident TBI status in associations of late‐life log_2_NfL (similar to our primary analysis population) and late‐life log_2_pTau181 with incident dementia (eFigure 8). In analyses evaluating interaction by TBI frequency, TBI severity, and timing of TBI, results were similar to those of our primary analysis population (eTable 15).

## 4 DISCUSSION

In this community‐based cohort, levels of neuronal degeneration (NfL) and astrogliosis (GFAP) biomarkers remained elevated for 12 to 14 years after TBI, and AD‐related plasma biomarkers (inverted Aβ_42_/Aβ_40_ ratio and pTau181) remained elevated for 8 to 9 years after TBI. This study also provides evidence that TBI events may modify associations of plasma biomarkers with dementia risk, with the most consistent effect modification observed in associations of late‐life NfL with dementia risk. Taken together, our results provide evidence that TBI is associated with AD‐related biomarkers and more strongly associated with biomarkers of neuronal injury and astrogliosis, suggesting that the pathologic processes underlying post‐TBI dementia are heterogeneous, that individuals with preclinical changes in neurodegenerative biomarkers may be more susceptible to TBI (i.e., that associations are bi‐directional), or a combination thereof.

Our analyses examining trajectories of biomarkers extend the prior literature by providing robust estimates of biomarker trajectories over 26 years from midlife to late life and before and after TBI events. Importantly, this study design enabled us to estimate the time from injury to intersection with no/pre‐TBI biomarker trajectories. Consistent with prior work with shorter follow‐up (i.e., around 5 years),^3^, ^4^ we found that levels of NfL and GFAP remained elevated for years after injury. Our work suggests that levels of NfL and GFAP remain elevated for 12 to 14 years after injury on average, which may be indicative of ongoing axonal degradation with resultant white matter degeneration and astrogliosis.^6^ Similarly, our data on the inverted Aβ_42_/Aβ_40_ ratio are consistent with studies with shorter post‐injury follow‐up.^22^ In contrast to a study of individuals with moderate to severe TBI that did not find elevations in pTau181 in the first year after injury,^5^ we did observe elevations in pTau181 levels, but these elevations persisted for a shorter amount of time after the injury (8 years) than the other plasma biomarkers evaluated. The persistent elevations in plasma biomarkers for years after the injury likely reflects the downstream neuropathological changes secondary to the injury, which eventually return to trajectories of pre‐injury neurodegenerative changes. That NfL and GFAP were elevated above pre‐injury trajectories for a longer length of time compared to the inverted Aβ_42_/Aβ_40_ ratio and pTau181 may suggest that post‐TBI processes are represented by axonal and astrogliosis pathology more than by AD‐related pathology.

The analyses investigating how TBI modifies associations of plasma biomarkers with dementia risk also provide mechanistic insights into post‐TBI dementia. We observed the most consistent effect modification by TBI status for associations of late‐life NfL with dementia risk, although the magnitude of additive interaction was modest. This observed non‐multiplicative, positive additive interaction suggests that individuals with TBI may be more susceptible to neuronal degeneration (i.e., elevated NfL) in terms of dementia risk, not because NfL increases the relative risk more (i.e., non‐multiplicative interaction), but because individuals with TBI have higher baseline risk of dementia (i.e., positive additive interaction) – this means that the same relative risk translates into a larger absolute increase in risk of dementia associated with NfL among individuals with versus without TBI. In our secondary analyses, there was evidence of both additive and multiplicative interaction by TBI frequency and timing of injury for midlife Aβ_42_/Aβ_40_ ratio and late‐life NfL and pTau181 with dementia risk, although sample sizes were limited in these analyses. Taken together, these findings are consistent with *post mortem* pathology studies that suggest that post‐TBI neurodegeneration is multifactorial. It is likely that both pre‐injury pathologic processes (which may be subclinical/latent) and pathologic processes triggered de novo by trauma contribute to associations of TBI with dementia.^23^, ^24^

Our results should be interpreted in the context of limitations. First, self‐reported TBI is defined using a non‐validated self‐report questionnaire. However, the self‐report questions consistently inquire about injuries associated with loss of consciousness and/or the need for medical care. These self‐report data are combined with ICD‐code data to define TBI, so there remains the possibility of misclassification of individuals who sustained a milder TBI but did not seek medical care, which may have resulted in an underestimation of associations. Consistent with this, the cumulative incidence of TBI over 26 years (13%) in ARIC is slightly lower than national estimates of 16% to 19%.^25^ Additionally, secondary analyses of TBI frequency and severity had lower statistical power and increased risk of model overfitting due to the smaller subgroup sample sizes. It is also important to note that the results of our primary analyses may not generalize to individuals who sustained a TBI prior to midlife, as our primary analytic population included individuals without TBI occurring prior to the first biomarker measurement in midlife. This study design likely contributes to the relatively older onset of dementia among individuals with versus without TBI, which is in contrast to the prior literature, which investigated lifetime TBI status.^26^, ^27^ Second, the plasma biomarkers were measured at up to three time points (1993 to 1995, 2011 to 2013, and 2016 to 2019) over a period of 26 years, limiting more precise estimates of trajectories and the ability to model any potential non‐linearities in the data. Further, our study had data on one pTau isoform (pTau181), but other isoforms, such as pTau217 and pTau231, have shown greater promise in relation to dementia risk assessment.^28^ Third, while the ARIC study dementia definition is not dependent on visit attendance, which minimizes potential biases due to the competing risk of mortality and due to study attrition, it is a measure of all‐cause dementia and cause‐specific dementia diagnoses, and neuropathological data were not available.

In conclusion, this study provides evidence that TBI alters trajectories of plasma biomarkers of neuronal degeneration, astrogliosis, and, to a lesser extent, AD‐related pathology, for approximately a decade after injury. Further, our findings also suggest that an incident TBI event adds to and interacts with ongoing neurodegenerative processes to increase the risk of later‐life dementia.

## CONFLICT OF INTEREST STATEMENT

The authors declare no conflicts of interest relevant to this work. Author disclosures are available in the supporting information.

## CONSENT STATEMENT

The ARIC study is approved by Institutional Review Boards at all participating institutions, and written informed consent was obtained from each participant or their legally authorized representative at study visits.

## Supporting information



Supporting Information

Supporting Information
