# Supplementary material for: Traumatic brain injury, changes in plasma amyloid, tau, and neurodegenerative biomarkers, and dementia risk
Source: Alzheimers Dement. 2025 Sep 15;21(9):e70611. doi: 10.1002/alz.70611 (PMC12434701; doi:10.1002/alz.70611)
Supplement: Supplementary file 2 — Supporting Information [file ALZ-21-e70611-s001.docx]

**Supplemental Materials**

**Table of Contents**

[**eMethods** 3](#_Toc203139106)

[**Plasma Biomarkers** 3](#_Toc203139107)

[**Covariates** 3](#_Toc203139108)

[**Mixed Effects Models** 3](#_Toc203139109)

[**Multiple Imputation** 4](#_Toc203139110)

[**Inverse Probability Weighting** 4](#_Toc203139111)

[**eTable 1. Characteristics of Atherosclerosis Risk in Communities (ARIC) Cohort Stratified by Inclusion in Primary Analysis Population of Change in Plasma Biomarkers Over Time (N=6,538)** 5](#_Toc203139112)

[**eTable 2. Characteristics of Atherosclerosis Risk in Communities (ARIC) Cohort Stratified by Inclusion in Primary Analysis Population of Association of Plasma Biomarkers and Incident Dementia (N=6,538)** 6](#_Toc203139113)

[**eTable 3. Definition of Traumatic Brain Injury (TBI) from Self-Report Questions and ICD-9/10 Codes** 7](#_Toc203139114)

[**eTable 4.** **Test-Retest Reliability Estimates and Minimal Detectable Change of Plasma Biomarkers in Midlife Measured Using the Quanterix Simoa Platform, the ARIC Study, Visit 3 (1993-1995, N=38)** 8](#_Toc203139115)

[**eTable 5.** **Test-Retest Reliability Estimates and Minimal Detectable Change of Plasma Biomarkers in Late-Life Measured Using the Quanterix Simoa Platform, the ARIC Study, Visit 5 (2011-2013, N=90)** 9](#_Toc203139116)

[**eTable 6. Dementia Definition** 10](#_Toc203139117)

[**eTable 7. Age, Sex, Race, Education, Apolipoprotein ε4 Genotype, and Cognitive Diagnosis Stratified Change (95% CI) in Standardized Plasma Biomarkers by Any Traumatic Brain Injury (TBI) Status, the ARIC Study 1990-2019 (N=1,150)** 11](#_Toc203139118)

[**eTable 8. Covariate-Adjusted Change (95% CI) in** **Standardized Plasma Biomarkers by Self-Reported or ICD-9/10 Code Identified Traumatic Brain Injury (TBI) Status, the ARIC Study 1993-2019 (N=1,150)** 12](#_Toc203139119)

[**eTable 9. Characteristics of Sensitivity Analysis Population of Change in Plasma Biomarkers Over Time Stratified by Timing of Incident Traumatic Brain Injury (TBI) Status (N=1,501)** 13](#_Toc203139120)

[**eTable 10. Sensitivity Analysis of Covariate-Adjusted Change (95% CI) in Standardized Plasma Biomarkers by Traumatic Brain Injury (TBI) Status, the ARIC Study 1993-2019 (N=1,501)** 14](#_Toc203139121)

[**eTable 11. Characteristics of Primary Analysis Population of Association of Plasma Biomarkers and Incident Dementia Stratified by Incident Traumatic Brain Injury (TBI) Status (N=1,047)** 15](#_Toc203139122)

[**eTable 12. Covariate-Adjusted Association of Standardized Plasma Biomarkers with Incident Dementia in Late-Life by Traumatic Brain Injury (TBI) Frequency, Severity, and Timing of TBI, the ARIC Study 2011-2020 (N=1,047)** 16](#_Toc203139123)

[**eTable 13. Covariate-Adjusted Association of Standardized Plasma Biomarkers with Incident Dementia in Late-Life by Self-Reported or ICD-9/10 Code Identified Traumatic Brain Injury (TBI) Status, the ARIC Study 2011-2020 (N=1,047)** 17](#_Toc203139124)

[**eTable 14. Characteristics of Sensitivity Analysis Population of Association of Plasma Biomarkers and Incident Dementia Stratified by Timing of Incident Traumatic Brain Injury (TBI) Status (N=1,375)** 18](#_Toc203139125)

[**eTable 15. Sensitivity Analysis of Covariate-Adjusted Association of Standardized Plasma Biomarkers with Incident Dementia in Late-Life by Traumatic Brain Injury (TBI) Status, the ARIC Study 2011-2020 (N=1,375)** 19](#_Toc203139126)

[**eFigure 1. Study Timeline and Participant Follow-up Patterns, the ARIC Study 1990-2019** 20](#_Toc203139127)

[**eFigure 2. Covariate-Adjusted, Model-Based Estimates of Change in Plasma Biomarkers Over Time by Traumatic Brain Injury (TBI) Frequency, the ARIC Study 1993-2019 (N=1,150)** 21](#_Toc203139128)

[**eFigure 3. Covariate-Adjusted, Model-Based Estimates of Change in Plasma Biomarkers Over Time by Traumatic Brain Injury (TBI) Severity, the ARIC Study 1993-2019 (N=1,131)** 22](#_Toc203139129)

[**eFigure 4. Covariate-Adjusted, Model-Based Estimates of Change in Plasma Biomarkers Over Time by Self-Reported Traumatic Brain Injury (TBI) Status, the ARIC Study 1993-2019 (N=1,150)** 23](#_Toc203139130)

[**eFigure 5. Covariate-Adjusted, Model-Based Estimates of Change in Plasma Biomarkers Over Time by ICD-9/10 Code Identified Traumatic Brain Injury (TBI) Status, the ARIC Study 1993-2019 (N=1,150)** 24](#_Toc203139131)

[**eFigure 6. Sensitivity Analysis of Covariate-Adjusted Change (95% CI) in Standardized Plasma Biomarkers by Traumatic Brain Injury (TBI) Status, Excluding Biomarker Observations Occurring within 1-Year Post-TBI, the ARIC Study 1993-2019 (N=1,150)** 25](#_Toc203139132)

[**eFigure 7. Sensitivity Analysis of Covariate-Adjusted Association of Standardized Plasma Biomarkers with Incident Dementia in Late-Life by Any Traumatic Brain Injury (TBI) Status, Excluding Biomarker Observations Occurring within 1-Year Post-TBI, the ARIC Study 2011-2020 (N=1,047)** 27](#_Toc203139133)

[**eFigure 8. Sensitivity Analysis of Covariate-Adjusted Association of Standardized Plasma Biomarkers with Incident Dementia in Late-Life by Any Traumatic Brain Injury (TBI) Status, the ARIC Study 2011-2020 (N=1,375)** 28](#_Toc203139134)

[**eReferences** 29](#_Toc203139135)

# **eMethods**

## **Plasma Biomarkers**

At each clinic-based visit,^1^ ethylenediaminetetraacetic acid whole-blood samples were collected and placed in an ice bath until plasma was separated by centrifugation. Plasma was divided into aliquots in 1.5-mL tubes, frozen, and stored in -80°C freezers. Plasma was thawed immediately before being aliquoted, plated, and analyzed by HD-X instruments that measured multiple biomarkers simultaneously using the single molecule array technology (Simoa) Neurology 4-Plex E (N4PE) from Quanterix (Billerica, MA). Simoa is an ultrasensitive, digital immunoassay platform that can detect proteins in plasma at sub-femtomolar concentrations.^2,3^ The N4PE assay included measures of amyloid β isoforms that were 40 (Aβ40) or 42 (Aβ42) residues long and measured the accumulation of extracellular amyloid plaques, neurofilament light (NfL) which quantified axonal injury, and glial fibrillary acidic protein (GFAP) which quantified inflammation and astrocytic activation. A singleplex assay for phosphorylated tau at threonine 181 (p-Tau181, version 1.0) measured intracellular neurofibrillary tangles.

To evaluate test-retest reliability,^4^ two plasma aliquots were obtained from the same participant in a subsample in midlife (Visit 3, 1993-95, N=38) and late-life (Visit 5, 2011-13 N=90). The plasma samples were blindly analyzed by the Advanced Research and Diagnostic Laboratory at the University of Minnesota using the same reagent batch. The lower detection limits of the Simoa N4PE assay were 1.54 pg/mL for Aβ40, 0.54 pg/mL for Aβ42, 0.36 pg/mL for NfL, and 1.76 pg/mL for GFAP. The lower detection limit of the singleplex p-Tau181 assay was 0.11 pg/mL. The upper detection limits of the Simoa N4PE assay were 336 pg/mL for Aβ40, 89.6 pg/mL for Aβ42, 1,376 pg/mL for NfL, and 18,584 pg/mL for GFAP. The upper detection limit of the singleplex p-Tau181 assay was 351.2 pg/mL. Among the midlife assays included in the primary analysis population of change in plasma biomarkers over time (N=1,150), 2 p-Tau181 assays and 21 Aβ40 or Aβ42 assays were beyond the limits of detection and set at the minimum or maximum detectable value. Among late-life assays in the same analysis population (N=1,150), only 3 Aβ40 or Aβ42 assays were beyond the limits of detection. The correlation and agreement between aliquots from the same participants were quantified by fitting a linear mixed effects model that estimated between-participant and within-participant variance. The intraclass correlation coefficient was calculated by dividing between-participant variance by the total variance. 95% confidence intervals (95% CI) were generated using percentile bootstrapping with 10,000 samples. The intraclass correlation coefficient was acceptable in midlife (0.75-0.93) and excellent in late-life (0.84-0.95) (**eTables 4 and 5**). The precision and repeatability of the assays was quantified by calculating the coefficient of variation as the average of each within-participant ratio of the standard deviation to the mean. The coefficient of variation was acceptable in midlife (3.2-20.7) and excellent in late-life (2.3-11.3). Within-participant error was quantified as the standard error of measurement, which was subsequently used to calculate minimal detectable change with 95% confidence. Minimal detectable change indicated the amount of change that could be reliably detected given measurement error.

**Covariates**

Time invariant covariates (confounders) were selected *a priori* and include age, sex, race/center, and education. Date of birth was self-reported at Visit 1 and used to calculate age (years). Self-reported sex (male; female) and race (Asian; American Indian/Alaskan Indian; Black; White) were collected at Visit 1 and a combined race/center covariate (White, Forsyth County, NC; Black, Forsyth County, NC; White, Minneapolis suburbs, MN; White, Washington County, MD; Black, Jackson, MS) was used in analyses due to race/center aliasing resulting in insufficient numbers of Asian and American Indian/Alaskan Indian participants and insufficient numbers of Black participants in Maryland and Minnesota. Education was self-reported at Visit 1 (less than high school; high school, GED, or vocational school; some college, graduate, or professional school). To reduce measurement bias, time-varying covariates included creatinine-cystatin C estimated glomerular filtration rate^5^ (eGFR; mL/min/1.73m^2^) and body mass index (BMI; kg/m^2^).^4,6,7^

**Mixed Effects Models**

The two-level linear mixed effects models that estimated the association of time-varying incident TBI with change in plasma biomarkers comprised repeated assessments (level 1, *j*) nested within participants (level 2, *i*).

*Y_ij_ = α_0i_ + (β_1_+α_1i_)*Time_ij_ + β_2_*TBI_ij_ + β_3_*Time_ij_*TBI_ij_ + β_4_*Cov_ij_ + β_5_*Cov_ij_*Time_ij_ + ε_ij_*

The model included a random intercept (*α_0i_*) and a random slope (*α_1i_*) for the time between the baseline assessment and subsequent follow-up assessments *(β_1_*Time_ij_*). Incident TBI (*β_2_*TBI_ij_*) was 0 for all participants at the baseline assessment and 1 at follow-up assessments if incident TBI occurred before the follow-up assessment. An interaction was specified between time and TBI (*β_3_*Time_ij_*TBI_ij_*). Time-invariant and time-varying covariates (*β_4_*Cov_ij_*) were included in the model and an interaction was specified between each time-invariant covariate and time (*β_5_*Cov_ij_*Time_ij_*). Model coefficients of interest included the following:

1. *β_1_* which estimated change per decade in biomarker levels among individuals without TBI.
2. *β_1_ + β_3_* which estimated change per decade in biomarker levels among individuals after incident TBI.
3. *β_3_* which estimated the difference in the change per decade in biomarker levels between individuals with and without TBI.
4. *β_2_* *+ β_3_* which estimated the difference in biomarker levels following incident TBI at a select point in time.

Since a specific time had to be chosen for the estimate of *β_2_* *+ β_3_* and none of the participants had incident TBI at the baseline, the median time of incident TBI time was used (12.8 years post-Visit 3, 1993-1995). For models examining TBI frequency, the second TBI injury was specified as occurring at the median time of the second TBI (19.0 years post-Visit 3, 1993-1995).

**Multiple Imputation**

Missing covariates were imputed using multiple imputation by chained equations^8^ with auxiliary variables.^9,10^ The imputation model included all covariates previously described plus age squared and time-varying measures of total cholesterol, high density lipoprotein (HDL) cholesterol, hypertension, diabetes, hospitalizations, coronary heart disease, cognitive function, cigarette use, and alcohol use. Ten imputed datasets were generated based on an analysis^11^ indicating that precision would be optimal if at least six imputed datasets were analyzed. Parameter estimates from models fit to imputed data were combined according to Rubin’s rules.^12^

Total cholesterol was determined from plasma samples using enzymatic methods.^13,14^ A Beckman Olympus AU400 Series (Olympus, Tokyo, Japan) chemistry analyzer calculated total cholesterol and HDL cholesterol. Systolic (SBP) and diastolic (DBP) blood pressure were measured by the Omron HEM-907 XL oscillometric automated sphygmomanometer (Omron Healthcare, Kyoto, Japan). Hypertension was defined as present when SBP ≥140 mm/Hg, DBP ≥90 mm/Hg, or the participant reported the use of anti-hypertensive medication. Diabetes was defined as present by fasting glucose ≥ 126 mg/dL, non-fasting glucose ≥ 200 mg/dL, the use of glucose-lowering medication, or self-reported physician diagnosis of diabetes. Hospitalizations were determined from medical records.^17^ Adjudication of medical records identified cases of coronary heart disease (CHD).^15,16^ At Visit 2 (1990-1992) and Visit 4 (1996-1998), cognitive function was measured using the Word Fluency Test,^17^ Digit Symbol Substitution,^18^ and Delayed Word Recall.^19^ During Visits 5 through 7, the cognitive battery was expanded to include Digit Span Backwards,^18^ Boston Naming Test,^20^ Animal Naming Score,^17^ Trail Making Tests A and B,^21^ Incidental Learning,^22^ and the Logical Memory Test.^18^ Available cognitive tests at each visit were used to compute a factor score of global cognitive function for each participant.^23^ At Visit 5, each participant also completed the Mini Mental State Exam^24^ which was added to the imputation model as an auxiliary variable. Cigarette use (current, former, never) and alcohol use (current, former, never) were ascertained by self-report.

**Inverse Probability Weighting**

To mitigate bias introduced by selecting ARIC participants who previously enrolled in a neuroimaging substudy at Visit 5, sampling weights were computed as the product of inverse sampling fractions and the inverse probability of enrollment in the substudy.^25^ Applying these weights reduced selection bias conditional on the assumption that eligible participants who enrolled in the substudy were representative of eligible participants who did not enroll.

To reduce attrition bias in mixed effects models that examined change in plasma biomarkers over time, stabilized inverse probability of attrition weights conditional on being alive^26,27^ were calculated using a logistic regression model that included all measures utilized in the imputation model. Time-varying attrition weights were applied to each visit (Level 1) and time-invariant sampling weights were applied to each participant (Level 2). Integrating these weights into linear mixed effects models reduced bias from informative attrition under the assumption that missingness was random conditional on covariates in the logistic regression model.

# **eTable 1. Characteristics of Atherosclerosis Risk in Communities (ARIC) Cohort Stratified by Inclusion in Primary Analysis Population of Change in Plasma Biomarkers Over Time (N=6,538)**

|  | **Overall** | **Included (N=1,150)** | **Excluded (N=5,388)** |
| --- | --- | --- | --- |
| **Visit 5 (2011-13)** Age, years | 76.3 (5.3) [6538] | 76.8 (5.4) [1150] | 76.2 (5.2) [5388] |
| Female sex | 3845 (59%) [6538] | 757/1150 (66%) | 3088/5388 (57%) |
| Race and center  White, Minneapolis suburbs, Minnesota | 1901/6496 (29%) | 267/1150 (23%) | 1634/5346 (31%) |
| White, Washington County, Maryland | 1751/6496 (27%) | 305/1150 (27%) | 1446/5346 (27%) |
| Black, Jackson, Mississippi | 1416/6496 (22%) | 309/1150 (27%) | 1107/5346 (21%) |
| White, Forsyth County, North Carolina | 1325/6496 (20%) | 247/1150 (22%) | 1078/5346 (20%) |
| Black, Forsyth County, North Carolina | 103/6496 (2%) | 22/1150 (2%) | 81/5346 (2%) |
| Education  Less than completed high school | 990/6527 (15%) | 154/1150 (13%) | 836/5377 (16%) |
| High school, GED, or vocational school | 2713/6527 (42%) | 481/1150 (42%) | 2232/5377 (42%) |
| Some college, graduate, or professional school | 2824/6527 (43%) | 515/1150 (45%) | 2309/5377 (44%) |
| One or more apolipoprotein E alleles | 1815/6296 (29%) | 304/1088 (28%) | 1511/5208 (29%) |
| Cigarette use  Current | 364/5778 (6%) | 58/1150 (5%) | 306/4628 (7%) |
| Former | 2993/5778 (52%) | 559/1150 (49%) | 2434/4628 (53%) |
| Never | 2421/5778 (42%) | 533/1150 (46%) | 1888/4628 (41%) |
| Alcohol use  Current | 2980/6108 (49%) | 510/1119 (46%) | 2470/4989 (50%) |
| Former | 1816/6108 (30%) | 332/1119 (30%) | 1484/4989 (30%) |
| Never | 1312/6108 (22%) | 277/1119 (25%) | 1035/4989 (21%) |
| Diabetes | 2126/6286 (34%) | 361/1150 (31%) | 1765/5136 (34%) |
| Hypertension | 4905/6457 (76%) | 955/1150 (83%) | 3950/5307 (74%) |
| Body mass index, kg/m^2^ | 28.7 (5.8) [6269] | 28.3 (5.3) [1145] | 28.9 (5.9) [5124] |
| Estimated glomerular filtration rate, mL/min/1.73m^2^ | 68.3 (19.3) [6437] | 69.7 (17.3) [1149] | 68.0 (19.7) [5288] |
| TBI Frequency  No TBI | 4401/6515 (68%) | 998/1150 (87%) | 3403/5365 (63%) |
| 1 TBI | 675/6515 (10%) | 78/1150 (7%) | 597/5365 (11%) |
| 2+ TBIs | 1439/6515 (22%) | 74/1150 (6%) | 1365/5365 (25%) |
| TBI Severity  No TBI | 4699/5518 (85%) | 998/1131 (88%) | 3701/4387 (84%) |
| Mild TBI | 656/5518 (12%) | 99/1131 (9%) | 557/4387 (13%) |
| Moderate, severe, penetrating TBI | 166/5518 (3%) | 34/1131 (3%) | 132/4387 (3%) |
| Dementia on or before 2020 | 1572/6538 (24%) | 300 (26%) [1150] | 1272/5388 (24%) |
| Death on or before 2020 | 1968/6538 (30%) | 313 (27%) [1150] | 1655/5388 (31%) |

Abbreviations: GED, General Educational Development credential; kg, kilogram; m^2^, meters squared; min, minute; mL.

**Data are n (%) or mean (SD**) [total]**. Denominators for percentages are based on the number of participants with complete data.**

# **eTable 2. Characteristics of Atherosclerosis Risk in Communities (ARIC) Cohort Stratified by Inclusion in Primary Analysis Population of Association of Plasma Biomarkers and Incident Dementia (N=6,538)**

|  | **Overall** | **Included (N=1,047)** | **Excluded (N=5,491)** |
| --- | --- | --- | --- |
| **Visit 5 (2011-13)** Age, years | 76.3 (5.3) [6538] | 76.6 (5.3) [1047] | 76.3 (5.3) [5491] |
| Female sex | 3845/6538 (59%) | 690/1047 (66%) | 3155/5491 (58%) |
| Race and center  White, Minneapolis suburbs, Minnesota | 1901/6496 (29%) | 243/1047 (23%) | 1658/5449 (30%) |
| White, Washington County, Maryland | 1751/6496 (27%) | 279/1047 (27%) | 1472/5449 (27%) |
| Black, Jackson, Mississippi | 1416/6496 (22%) | 286/1047 (27%) | 1130/5449 (21%) |
| White, Forsyth County, North Carolina | 1325/6496 (20%) | 222/1047 (21%) | 1103/5449 (20%) |
| Black, Forsyth County, North Carolina | 103/6496 (2%) | 17/1047 (2%) | 86/5449 (2%) |
| Education  Less than completed high school | 990/6527 (15%) | 133/1047 (13%) | 857/5480 (16%) |
| High school, GED, or vocational school | 2713/6527 (42%) | 437/1047 (42%) | 2276/5480 (42%) |
| Some college, graduate, or professional school | 2824/6527 (43%) | 477/1047 (46%) | 2347/5480 (43%) |
| One or more apolipoprotein E alleles | 1815/6296 (29%) | 267/998 (27%) | 1548/5298 (29%) |
| Cigarette use  Current | 364/5778 (6%) | 54/1047 (5%) | 310/4731 (7%) |
| Former | 2993/5778 (52%) | 511/1047 (49%) | 2482/4731 (53%) |
| Never | 2421/5778 (42%) | 482/1047 (46%) | 1939/4731 (41%) |
| Alcohol use  Current | 2980/6108 (49%) | 471/1020 (46%) | 2509/5088 (49%) |
| Former | 1816/6108 (30%) | 303/1020 (30%) | 1513/5088 (30%) |
| Never | 1312/6108 (22%) | 246/1020 (24%) | 1066/5088 (21%) |
| Diabetes | 2126/6286 (34%) | 326/1047 (31%) | 1800/5239 (34%) |
| Hypertension | 4905/6457 (76%) | 870/1047 (83%) | 4035/5410 (75%) |
| Body mass index, kg/m^2^ | 28.7 (5.8) [6269] | 28.3 (5.2) [1043] | 28.8 (5.9) [5226] |
| Estimated glomerular filtration rate, mL/min/1.73m^2^ | 68.3 (19.3) [6437] | 70.4 (17.2) [1046] | 67.9 (19.6) [5391] |
| TBI Frequency  No TBI | 4401/6515 (68%) | 909/1047 (87%) | 3492/5468 (64%) |
| 1 TBI | 675/6515 (10%) | 72/1047 (7%) | 603/5468 (11%) |
| 2+ TBIs | 1439/6515 (22%) | 66/1047 (6%) | 1373/5468 (25%) |
| TBI Severity  No TBI | 4699/5521 (85%) | 909/1034 (88%) | 3790/4487 (84%) |
| Mild TBI | 656/5521 (12%) | 93/1034 (9%) | 563/4487 (13%) |
| Moderate, severe, penetrating TBI | 166/5521 (3%) | 32/1034 (3%) | 134/4487 (3%) |
| Dementia on or before 2020 | 1572/6538 (24%) | 237/1047 (23%) | 1335/5491 (24%) |
| Death on or before 2020 | 1968/6538 (30%) | 235/1047 (22%) | 1733/5491 (32%) |

Abbreviations: GED, General Educational Development credential; kg, kilogram; m^2^, meters squared; min, minute; mL.

**Data are n (%) or mean (SD**) [total]. **Denominators for percentages are based on the number of participants with complete data.**

# **eTable 3. Definition of Traumatic Brain Injury (TBI) from Self-Report Questions and ICD-9/10 Codes**

| **Self-Report Questions**  ARIC Visit 3 (1993-95)   - Have you ever had a head injury which led you to see a physician or seek hospital care? - How many times has this happened? - How many of these head injuries resulted in your losing consciousness, no matter how briefly? - In what year was your head injury for which you sought medical care?   ARIC Visit 4 (1996-1998)   - Have you ever had a major head injury? That is, one that resulted in your losing consciousness, no matter how briefly, or that led you to see a physician or seek hospital care? - How many times has this happened? - How many head injuries resulted in your losing consciousness, no matter how briefly? - In what year was your head injury for which you lost consciousness sought medical care?   ARIC Brain MRI Visit (2004-2006)*   - Have you ever had a head injury that resulted in loss of consciousness (knocked out)? - How many times? - In what year or how old were you when this first occurred? - In what year or how old were you when this last occurred?   ARIC Visit 5 (2011-13)*, ARIC Visit 6 (2016-2017), and ARIC Visit 7 (2018-2019)   - Have you ever had a head injury that resulted in loss of consciousness? - Have you had a head injury with extended loss of consciousness (>5 minutes)? - Have you had a head injury that resulted in long-term problems or dysfunction? | **ICD-9 Codes**   - 800.xx Fracture of vault of skull - 801.xx Fracture of base of skull - 803.xx Other and unqualified skull fractures - 804.xx Multiple fractures involving skull or face with other bones - 850.xx Concussion - 851.xx Cerebral laceration and contusion - 852.xx Subarachnoid, subdural, and extradural haemorrhage following injury - 853.xx Other and unspecified intracranial haemorrhage following injury - 854.xx Intracranial injury of other   **ICD-10 Codes**   - 959.01 Head injury, unspecified - S02.0 Fracture of vault of skull - S02.1X Fracture of base of skull - S02.8 Fractures of other unspecified skull and facial bones - S02.91 Unspecified fracture of skull - S04.02 Injury of optic chiasm - S04.03X Injury of optic tract and pathways - S04.04X Injury of visual cortex - S06.X Intracranial injuries, concussion, traumatic cerebral edema, diffuse and focal traumatic brain injury, traumatic epidural, subdural, and subarachnoid haemorrhage - S07.1 Crushing injury of skull |
| --- | --- |

*Questions asked in subgroup of ARIC participants selected for brain magnetic resonance imaging scans.

#

# **eTable 4. Test-Retest Reliability Estimates and Minimal Detectable Change of Plasma Biomarkers in Midlife Measured Using the Quanterix Simoa Platform, the ARIC Study, Visit 3 (1993-1995, N=38)**

|  | **Intraclass Correlation Coefficient^a^ (95% CI)** | **Coefficient of Variation^b^** | **Standard Error of Measurement^c^** | **Minimal Detectable Change_95_** |
| --- | --- | --- | --- | --- |
| Amyloid Beta 42 | 0.82 (0.70, 0.91) | 18.4 | 0.64 | 1.78 |
| Amyloid Beta 40 | 0.84 (0.74, 0.92) | 17.6 | 10.14 | 28.10 |
| Phosphorylated Tau-181 | 0.90 (0.78, 0.96) | 20.7 | 0.25 | 0.69 |
| Neurofilament Light | 0.93 (0.85, 0.97) | 10.1 | 1.09 | 3.02 |
| Glial Fibrillary Acidic Protein | 0.91 (0.86, 0.95) | 13.7 | 13.47 | 37.34 |
| Aß42/Aß40 Ratio | 0.75 (0.50, 0.94) | 10.0 | 0.01 | 0.03 |
| Log2 Phosphorylated Tau-181 | 0.76 (0.58, 0.90) | 15.7 | 0.42 | 1.16 |
| Log2 Neurofilament Light | 0.89 (0.78, 0.95) | 5.0 | 0.18 | 0.50 |
| Log2 Glial Fibrillary Acidic Protein | 0.91 (0.85, 0.95) | 3.2 | 0.21 | 0.58 |

Abbreviations: _95_, 95% confidence; CI, confidence intervals.

^a^Correlation and agreement between plasma aliquots obtained from the same person was quantified by fitting a linear mixed effects model that estimated between-participant and within-participant variance. The intraclass correlation coefficient was calculated by dividing between-participant variance by the total variance. 95% confidence intervals were obtained via percentile bootstrapping with 10,000 samples.

^b^Precision and repeatability of the assays was quantified by calculating the coefficient of variation as the average of each within-participant ratio of the standard deviation to the mean.

^C^Within- and between-participant variance from the mixed effects model was used to calculate the standard error of measurement. Minimal detectable change denoting true change above measurement error with 95% confidence was calculated utilizing the standard error of measurement.

# **eTable 5. Test-Retest Reliability Estimates and Minimal Detectable Change of Plasma Biomarkers in Late-Life Measured Using the Quanterix Simoa Platform, the ARIC Study, Visit 5 (2011-2013, N=90)**

|  | **Intraclass Correlation Coefficient^a^ (95% CI)** | **Coefficient of Variation^b^** | **Standard Error of Measurement^c^** | **Minimal Detectable Change_95_** |
| --- | --- | --- | --- | --- |
| Amyloid Beta 42 | 0.87 (0.82, 0.92) | 7.3 | 0.54 | 1.50 |
| Amyloid Beta 40 | 0.84 (0.77, 0.90) | 8.5 | 9.87 | 27.35 |
| Phosphorylated Tau-181 | 0.93 (0.89, 0.96) | 9.7 | 0.33 | 0.92 |
| Neurofilament Light | 0.95 (0.93, 0.97) | 9.8 | 2.32 | 6.42 |
| Glial Fibrillary Acidic Protein | 0.91 (0.85, 0.95) | 11.3 | 23.45 | 65.01 |
| Aß42/Aß40 Ratio | 0.87 (0.76, 0.93) | 6.2 | 0.00 | 0.01 |
| Log2 Phosphorylated Tau-181 | 0.92 (0.87, 0.96) | 5.6 | 0.18 | 0.49 |
| Log2 Neurofilament Light | 0.91 (0.82, 0.96) | 3.7 | 0.20 | 0.55 |
| Log2 Glial Fibrillary Acidic Protein | 0.88 (0.78, 0.94) | 2.3 | 0.21 | 0.59 |

Abbreviations: _95_, 95% confidence; CI, confidence intervals.

^a^Correlation and agreement between plasma aliquots obtained from the same person was quantified by fitting a linear mixed effects model that estimated between-participant and within-participant variance. The intraclass correlation coefficient was calculated by dividing between-participant variance by the total variance. 95% confidence intervals were obtained via percentile bootstrapping with 10,000 samples.

^b^Precision and repeatability of the assays was quantified by calculating the coefficient of variation as the average of each within-participant ratio of the standard deviation to the mean.

^C^Within- and between-participant variance from the mixed effects model was used to calculate the standard error of measurement. Minimal detectable change denoting true change above measurement error with 95% confidence was calculated utilizing the standard error of measurement.

# **eTable 6. Dementia Definition**

|  |  | Dementia |
| --- | --- | --- |
| Level 1 | Attended Visit 5, 6, and/or 7 (all identified cases adjudicated by expert panel) | ≥2 cognitive domain Z-scores from current visit worse than -1.5 Z  *and*  Decline in z-score from the serial 3-test cognitive assessments (current score minus prior highest score) below 10^th^ percentile on one test or below 20^th^ percentile on two tests (Visit 5); Annualized decrease in global cognition factors score >-0.55 SD per year (Visit 6 and later visits)  *and*  Participant and/or Informant Clinical Dementia Rating Scale sum of boxes >3  *and*  Informant Functional Activities Questionnaire score >5  *OR*  Mini-Mental State Examination score <21 for whites or <19 for blacks^28,29^ |
| Level 2 | Alive, but did not attend Visit 5 but completed Visit 5 telephone assessment | Telephone Interview for Cognitive Status score ≤23 |
|  | Alive, but did not attend Visits 5, 6, and/or 7 *or* dead at time of Visits 5, 6, and/or 7 | Informant Clinical Dementia Rating Scale sum of boxes >3  *and*  Informant Functional Activities Questionnaire score >5 |
|  | Alive, semi-annual telephone follow-up after ARIC Visit 5 | Ascertain Dementia 8-item Informant Questionnaire score ≥2  *or*  Any two Six-Item Screener scores ≤3 or one or more impaired Six-Item Screener scores where only a single score is available due to participant death or withdrawing from the study |
| Level 3 | Surveillance over entire study duration | Hospitalization ICD-9/10 codes for dementia  *or*  Death certificate code for dementia |

# **eTable 7. Age, Sex, Race, Education, Apolipoprotein ε4 Genotype, and Cognitive Diagnosis Stratified Change (95% CI) in** **Standardized Plasma Biomarkers by Any Traumatic Brain Injury (TBI) Status, the ARIC Study 1990-2019 (N=1,150)**

|  |  | **Aß42/Aß40 Ratio^** | | **Log2 p-Tau181** | | **Log2 NfL** | | **Log2 GFAP** | |
| --- | --- | --- | --- | --- | --- | --- | --- | --- | --- |
|  |  | **Change Per Decade** | **Difference** | **Change Per Decade** | **Difference** | **Change Per Decade** | **Difference** | **Change Per Decade** | **Difference** |
|  | **N** | **ß (95% CI)** | **ß (95% CI)** | **ß (95% CI)** | **ß (95% CI)** | **ß (95% CI)** | **ß (95% CI)** | **ß (95% CI)** | **ß (95% CI)** |
| **Median Baseline Age** |  |  |  |  |  |  |  |  |  |
| Above 58.6 Years Old | 584 |  |  |  |  |  |  |  |  |
| No TBI (N=491) |  | 0.045 (0.000, 0.091) | Referent | 0.172 (0.089, 0.255) | Referent | 0.196 (0.122, 0.270) | Referent | 0.161 (0.085, 0.237) | Referent |
| TBI (N=93) |  | -0.069 (-0.178, 0.039) | -0.115 (-0.230, 0.001) | 0.040 (-0.168, 0.248) | -0.132 (-0.351, 0.087) | 0.057 (-0.129, 0.244) | -0.139 (-0.335, 0.058) | 0.039 (-0.149, 0.227) | -0.122 (-0.319, 0.075) |
| Below 58.6 Years Old | 566 |  |  |  |  |  |  |  |  |
| No TBI (N=507) |  | 0.036 (0.001, 0.072) | Referent | 0.158 (0.104, 0.213) | Referent | 0.239 (0.190, 0.289) | Referent | 0.211 (0.162, 0.261) | Referent |
| TBI (N=59) |  | -0.088 (-0.215, 0.039) | -0.124 (-0.254, 0.006) | -0.111 (-0.290, 0.069) | -0.269 (-0.456, -0.082) | 0.130 (-0.031, 0.291) | -0.109 (-0.278, 0.060) | -0.011 (-0.170, 0.148) | -0.223 (-0.389, -0.056) |
| **Sex** |  |  |  |  |  |  |  |  |  |
| Female | 757 |  |  |  |  |  |  |  |  |
| No TBI (N=648) |  | 0.045 (0.008, 0.082) | Referent | 0.138 (0.078, 0.198) | Referent | 0.227 (0.174, 0.280) | Referent | 0.179 (0.125, 0.233) | Referent |
| TBI (N=109) |  | -0.039 (-0.134, 0.056) | -0.085 (-0.182, 0.013) | -0.045 (-0.196, 0.106) | -0.183 (-0.339, -0.027) | 0.055 (-0.080, 0.189) | -0.172 (-0.311, -0.034) | -0.053 (-0.188, 0.081) | -0.232 (-0.371, -0.094) |
| Male | 393 |  |  |  |  |  |  |  |  |
| No TBI (N=350) |  | 0.026 (-0.022, 0.073) | Referent | 0.216 (0.136, 0.296) | Referent | 0.247 (0.172, 0.322) | Referent | 0.187 (0.115, 0.259) | Referent |
| TBI (N=43) |  | -0.137 (-0.327, 0.052) | -0.163 (-0.359, 0.033) | -0.091 (-0.406, 0.223) | -0.308 (-0.633, 0.018) | 0.338 (0.045, 0.631) | 0.091 (-0.213, 0.395) | 0.237 (-0.043, 0.518) | 0.050 (-0.241, 0.341) |
| **Race** |  |  |  |  |  |  |  |  |  |
| White | 819 |  |  |  |  |  |  |  |  |
| No TBI (N=694) |  | 0.034 (-0.002, 0.069) | Referent | 0.155 (0.094, 0.216) | Referent | 0.223 (0.170, 0.276) | Referent | 0.161 (0.106, 0.215) | Referent |
| TBI (N=125) |  | -0.069 (-0.154, 0.017) | -0.102 (-0.189, -0.016) | -0.055 (-0.204, 0.095) | -0.210 (-0.364, -0.056) | 0.103 (-0.028, 0.234) | -0.121 (-0.256, 0.015) | -0.041 (-0.174, 0.091) | -0.202 (-0.339, -0.065) |
| Black | 331 |  |  |  |  |  |  |  |  |
| No TBI (N=304) |  | 0.050 (-0.080, 0.181) | Referent | 0.371 (0.167, 0.576) | Referent | 0.473 (0.296, 0.650) | Referent | 0.465 (0.295, 0.634) | Referent |
| TBI (N=27) |  | -0.029 (-0.297, 0.240) | -0.079 (-0.330, 0.172) | 0.207 (-0.168, 0.582) | -0.164 (-0.515, 0.187) | 0.307 (-0.044, 0.657) | -0.166 (-0.497, 0.164) | 0.233 (-0.097, 0.563) | -0.232 (-0.543, 0.080) |
| **Education** |  |  |  |  |  |  |  |  |  |
| Less than high school | 154 |  |  |  |  |  |  |  |  |
| No TBI (N=137) |  | 0.011 (-0.070, 0.091) | Referent | 0.184 (-0.008, 0.377) | Referent | 0.122 (-0.067, 0.311) | Referent | 0.166 (0.006, 0.327) | Referent |
| TBI (N=17) |  | 0.016 (-0.140, 0.172) | 0.005 (-0.147, 0.158) | -0.040 (-0.384, 0.305) | -0.224 (-0.605, 0.157) | 0.006 (-0.337, 0.349) | -0.116 (-0.491, 0.259) | -0.050 (-0.340, 0.240) | -0.216 (-0.533, 0.101) |
| High school | 481 |  |  |  |  |  |  |  |  |
| No TBI (N=416) |  | 0.069 (0.015, 0.122) | Referent | 0.162 (0.085, 0.240) | Referent | 0.259 (0.186, 0.332) | Referent | 0.182 (0.114, 0.250) | Referent |
| TBI (N=65) |  | -0.016 (-0.190, 0.159) | -0.084 (-0.260, 0.092) | 0.123 (-0.122, 0.368) | -0.040 (-0.290, 0.211) | 0.252 (0.024, 0.480) | -0.007 (-0.241, 0.227) | 0.026 (-0.187, 0.239) | -0.156 (-0.374, 0.061) |
| Some college | 515 |  |  |  |  |  |  |  |  |
| No TBI (N=445) |  | 0.032 (-0.009, 0.074) | Referent | 0.167 (0.099, 0.235) | Referent | 0.226 (0.168, 0.284) | Referent | 0.200 (0.136, 0.264) | Referent |
| TBI (N=70) |  | -0.134 (-0.243, -0.024) | -0.166 (-0.279, -0.053) | -0.145 (-0.332, 0.042) | -0.312 (-0.503, -0.121) | 0.007 (-0.154, 0.168) | -0.219 (-0.384, -0.054) | -0.014 (-0.189, 0.161) | -0.214 (-0.393, -0.035) |
| **Apolipoprotein E** |  |  |  |  |  |  |  |  |  |
| 0 ε4 alleles | 784 |  |  |  |  |  |  |  |  |
| No TBI (N=681) |  | 0.036 (-0.001, 0.072) | Referent | 0.148 (0.093, 0.203) | Referent | 0.235 (0.185, 0.285) | Referent | 0.186 (0.136, 0.235) | Referent |
| TBI (N=103) |  | -0.086 (-0.183, 0.012) | -0.121 (-0.221, -0.022) | -0.036 (-0.178, 0.107) | -0.184 (-0.331, -0.037) | 0.071 (-0.061, 0.202) | -0.164 (-0.300, -0.029) | 0.004 (-0.124, 0.131) | -0.182 (-0.314, -0.050) |
| 1+ ε4 alleles | 333 |  |  |  |  |  |  |  |  |
| No TBI (N=290) |  | 0.049 (-0.004, 0.101) | Referent | 0.180 (0.082, 0.279) | Referent | 0.221 (0.137, 0.305) | Referent | 0.160 (0.072, 0.249) | Referent |
| TBI (N=43) |  | 0.042 (-0.169, 0.253) | -0.007 (-0.221, 0.208) | -0.141 (-0.565, 0.282) | -0.322 (-0.749, 0.106) | 0.377 (0.003, 0.751) | 0.156 (-0.222, 0.534) | -0.068 (-0.454, 0.318) | -0.228 (-0.617, 0.161) |
| **Cognitive Diagnosis** |  |  |  |  |  |  |  |  |  |
| Normal | 708 |  |  |  |  |  |  |  |  |
| No TBI (N=630) |  | 0.037 (0.000, 0.075) | Referent | 0.167 (0.109, 0.225) | Referent | 0.239 (0.188, 0.289) | Referent | 0.183 (0.132, 0.234) | Referent |
| TBI (N=78) |  | -0.095 (-0.210, 0.020) | -0.132 (-0.250, -0.015) | -0.039 (-0.214, 0.137) | -0.206 (-0.386, -0.025) | 0.085 (-0.069, 0.238) | -0.154 (-0.312, 0.004) | -0.038 (-0.190, 0.114) | -0.221 (-0.377, -0.065) |
| MCI or Dementia | 441 |  |  |  |  |  |  |  |  |
| No TBI (N=367) |  | 0.066 (0.010, 0.121) | Referent | 0.187 (0.094, 0.280) | Referent | 0.260 (0.168, 0.352) | Referent | 0.215 (0.124, 0.305) | Referent |
| TBI (N=74) |  | -0.030 (-0.153, 0.093) | -0.095 (-0.226, 0.036) | -0.032 (-0.241, 0.177) | -0.219 (-0.446, 0.007) | 0.109 (-0.099, 0.317) | -0.151 (-0.376, 0.073) | -0.015 (-0.217, 0.187) | -0.229 (-0.448, -0.011) |

Abbreviations: Aβ, amyloid-β; CI, confidence intervals; GFAP, glial fibrillary acidic protein; MCI, mild cognitive impairment; NfL, neurofilament light chain; p-tau181, phosphorylated Tau-181; TBI, traumatic brain injury.

^Indicates that the Aβ42/Aβ40 ratio was inverted so that higher values denote greater risk. For log2 p-tau181, log2 NfL, and log2 GFAP positive values indicate a faster rate of change and negative values indicate a slower rate.

The analytic sample was restricted to pre-dementia plasma biomarkers measured using the Quanterix Simoa platform. Parameter estimates generated from linear mixed effects models. TBI defined as occurring 12.8 years after Visit 3 (median time of injury). Biomarkers standardized to Visit 3 by mean-centring and dividing by 0.0459 for Aβ42/Aβ40 ratio, 0.8033 for log2 p-tau181, 0.6920 for log2 NfL, and 0.6867 for log2 GFAP. Models adjusted for age, sex, race-center, and education as time-invariant covariates and estimated glomerular filtration rate and body mass index as time-varying covariates. An interaction was specified between each time-invariant covariate and time. Multiple imputation by chained equations was employed to impute missing covariates. Inverse probability weighting was used to account for selection bias and informative attrition.

All p-values for interaction >0.1.

# **eTable 8. Covariate-Adjusted Change (95% CI) in Standardized Plasma Biomarkers by Self-Reported or ICD-9/10 Code Identified Traumatic Brain Injury (TBI) Status, the ARIC Study 1993-2019 (N=1,150)**

|  | **Difference at**  **Median Time of TBI** | **Change Per Decade** | **Difference** |
| --- | --- | --- | --- |
|  | **ß (95% CI)** | **ß (95% CI)** | **ß (95% CI)** |
|  | **Aß42/Aß40 Ratio^** | | |
| **Self-Reported TBI** |  |  |  |
| No TBI (N=1,093) | Referent | 0.031 (0.002, 0.060) | Referent |
| TBI (N=57) | 0.043 (-0.138, 0.224) | -0.024 (-0.170, 0.121) | -0.055 (-0.202, 0.091) |
| **ICD-9/10 Code Identified TBI** |  |  |  |
| No TBI (N=1,014) | Referent | 0.036 (0.007, 0.065) | Referent |
| TBI (N=136) | 0.120 (0.012, 0.229) | -0.075 (-0.166, 0.016) | -0.111 (-0.203, -0.019) |
|  | **Log2 p-Tau181** | | |
| **Self-Reported TBI** |  |  |  |
| No TBI (N=1,093) | Referent | 0.142 (0.096, 0.189) | Referent |
| TBI (N=57) | 0.110 (-0.170, 0.389) | 0.091 (-0.126, 0.309) | -0.051 (-0.271, 0.168) |
| **ICD-9/10 Code Identified TBI** |  |  |  |
| No TBI (N=1,014) | Referent | 0.153 (0.105, 0.200) | Referent |
| TBI (N=136) | 0.217 (0.037, 0.397) | -0.056 (-0.201, 0.089) | -0.209 (-0.357, -0.061) |
|  | **Log2 NfL** | | |
| **Self-Reported TBI** |  |  |  |
| No TBI (N=1,093) | Referent | 0.225 (0.183, 0.267) | Referent |
| TBI (N=57) | 0.249 (-0.001, 0.500) | 0.233 (0.042, 0.425) | 0.009 (-0.185, 0.202) |
| **ICD-9/10 Code Identified TBI** |  |  |  |
| No TBI (N=1,014) | Referent | 0.233 (0.190, 0.275) | Referent |
| TBI (N=136) | 0.234 (0.070, 0.398) | 0.108 (-0.022, 0.239) | -0.124 (-0.258, 0.009) |
|  | **Log2 GFAP** | | |
| **Self-Reported TBI** |  |  |  |
| No TBI (N=1,093) | Referent | 0.170 (0.128, 0.213) | Referent |
| TBI (N=57) | 0.140 (-0.120, 0.400) | 0.088 (-0.103, 0.280) | -0.082 (-0.276, 0.111) |
| **ICD-9/10 Code Identified TBI** |  |  |  |
| No TBI (N=1,014) | Referent | 0.181 (0.138, 0.224) | Referent |
| TBI (N=136) | 0.248 (0.080, 0.417) | -0.042 (-0.172, 0.089) | -0.222 (-0.355, -0.089) |

Abbreviations: Aβ, amyloid-β; CI, confidence intervals; GFAP, glial fibrillary acidic protein; ICD, international classification of diseases; NfL, neurofilament light chain; p-tau181, phosphorylated Tau-181; TBI, traumatic brain injury.

^Indicates that the Aβ42/Aβ40 ratio was inverted so that higher values denote greater risk. For log2 p-tau181, log2 NfL, and log2 GFAP positive values indicate a faster rate of change and negative values indicate a slower rate.

The analytic sample was restricted to pre-dementia plasma biomarkers measured using the Quanterix Simoa platform. Parameter estimates generated from linear mixed effects models. TBI defined as occurring 12.8 years after Visit 3 (median time of injury). Biomarkers standardized to Visit 3 by mean-centring and dividing by 0.0459 for Aβ42/Aβ40 ratio, 0.8033 for log2 p-tau181, 0.6920 for log2 NfL, and 0.6867 for log2 GFAP. Models adjusted for age, sex, race-center, and education as time-invariant covariates and estimated glomerular filtration rate and body mass index as time-varying covariates. An interaction was specified between each time-invariant covariate and time. Multiple imputation by chained equations was employed to impute missing covariates. Inverse probability weighting was used to account for selection bias and informative attrition.

# **eTable 9. Characteristics of Sensitivity Analysis Population of Change in Plasma Biomarkers Over Time Stratified by Timing of Incident Traumatic Brain Injury (TBI) Status (N=1,501)**

|  | **Overall** | **No TBI (N=998)** | **TBI Before Visit 3^a^ (N=351)** | **TBI After Visit 3 (N=152)** |
| --- | --- | --- | --- | --- |
| **Visit 3 (1993-95)** Age, years | 59.0 (5.1) [1501] | 58.9 (5.2) [998] | 58.7 (4.9) [351] | 60.2 (5.2) [152] |
| Female sex | 909/1501 (61%) | 648/998 (65%) | 152/351 (43%) | 109/152 (72%) |
| Race and center  White, Washington County, Maryland | 403/1501 (27%) | 255/998 (26%) | 98/351 (28%) | 50/152 (33%) |
| White, Minneapolis suburbs, Minnesota | 371/1501 (25%) | 223/998 (22%) | 104/351 (30%) | 44/152 (29%) |
| Black, Jackson, Mississippi | 359/1501 (24%) | 285/998 (29%) | 50/351 (14%) | 24/152 (16%) |
| White, Forsyth County, North Carolina | 342/1501 (23%) | 216/998 (22%) | 95/351 (27%) | 31/152 (20%) |
| Black, Forsyth County, North Carolina | 26/1501 (2%) | 19/998 (2%) | 4/351 (1%) | 3/152 (2%) |
| Education  Less than completed high school | 194/1500 (13%) | 137/998 (14%) | 4/3500 (11%) | 17/152 (11%) |
| High school, GED, or vocational school | 631/1500 (42%) | 416/998 (42%) | 150/350 (43%) | 65/152 (43%) |
| Some college, graduate, or professional school | 675/1500 (45%) | 445/998 (45%) | 160/350 (46%) | 70/152 (46%) |
| One or more apolipoprotein E alleles | 391/1420 (28%) | 264/945 (28%) | 87/332 (26%) | 40/143 (28%) |
| Cigarette use  Current | 175/1500 (12%) | 114/997 (11%) | 46/351 (13%) | 15/152 (10%) |
| Former | 598/1500 (40%) | 374/997 (38%) | 162/351 (46%) | 62/152 (41%) |
| Never | 727/1500 (49%) | 509/997 (51%) | 143/351 (41%) | 75/152 (49%) |
| Alcohol use  Current | 831/1499 (55%) | 514/997 (52%) | 236/351 (67%) | 81/151 (54%) |
| Former | 299/1499 (20%) | 205/997 (21%) | 62/351 (18%) | 32/151 (21%) |
| Never | 369/1499 (25%) | 278/997 (28%) | 53/351 (15%) | 38/151 (25%) |
| Diabetes | 117/1500 (8%) | 84/997 (8%) | 23/351 (7%) | 10/152 (7%) |
| Hypertension | 524/1501 (35%) | 371/998 (37%) | 102/351 (29%) | 51/152 (34%) |
| Body mass index, kg/m^2^ | 27.9 (5.0) [1501] | 27.9 (5.0) [998] | 27.7 (4.7) [351] | 28.4 (5.6) [152] |
| Estimated glomerular filtration rate, mL/min/1.73m^2^ | 91.6 (14.1) [1441] | 91.7 (13.8) [954] | 92.2 (14.4) [339] | 90.1 (15.4) [148] |
| **Plasma Assays** Amyloid-ß 42, pg/mL  Visit 3 (1993-95) | 3.81 (1.74) [1501] | 3.80 (1.78) [998] | 3.93 (1.61) [351] | 3.59 (1.68) [152] |
| Visit 5 (2011-13) | 6.39 (1.87) [1499] | 6.32 (1.89) [997] | 6.53 (1.79) [350] | 6.56 (1.85) [152] |
| Visit 6 & 7 (2016-19) | 6.72 (1.88) [527] | 6.65 (1.87) [343] | 6.82 (1.90) [148] | 7.02 (1.95) [36] |
| Amyloid-ß 40, pg/mL  Visit 3 (1993-95) | 59.9 (28.2) [1501] | 59.4 (28.5) [998] | 62.3 (26.7) [351] | 58.0 (29.6) [152] |
| Visit 5 (2011-13) | 109.0 (25.3) [1499] | 107.8 (25.4) [997] | 109.6 (25.0) [350] | 115.1 (24.5) [152] |
| Visit 6 & 7 (2016-19) | 112.8 (25.9) [527] | 112.1 (24.0) [343] | 113.7 (30.5) [148] | 114.9 (23.5) [36] |
| Aß42/Aß40 ratio  Visit 3 (1993-95) | 0.072 (0.049) [1501] | 0.073 (0.046) [998] | 0.071 (0.057) [351] | 0.073 (0.045) [152] |
| Visit 5 (2011-13) | 0.059 (0.014) [1499] | 0.059 (0.014) [997] | 0.060 (0.012) [350] | 0.058 (0.013) [152] |
| Visit 6 & 7 (2016-19) | 0.060 (0.012) [527] | 0.060 (0.013) [343] | 0.060 (0.011) [148] | 0.061 (0.009) [36] |
| Phosphorylated tau-181   Visit 3 (1993-95) | 1.60 (0.98) [1500] | 1.56 (0.94) [997] | 1.68 (1.02) [351] | 1.65 (1.09) [152] |
| Visit 5 (2011-13) | 3.20 (1.84) [1498] | 3.20 (1.83) [995] | 3.14 (1.83) [351] | 3.35 (1.96) [152] |
| Visit 6 & 7 (2016-19) | 3.34 (1.62) [527] | 3.31 (1.65) [343] | 3.37 (1.46) [148] | 3.51 (1.93) [36] |
| Log2 phosphorylated tau-181  Visit 3 (1993-95) | 0.47 (0.79) [1500] | 0.44 (0.78) [997] | 0.55 (0.76) [351] | 0.45 (0.93) [152] |
| Visit 5 (2011-13) | 1.50 (0.68) [1498] | 1.50 (0.68) [995] | 1.47 (0.70) [351] | 1.57 (0.70) [152] |
| Visit 6 & 7 (2016-19) | 1.59 (0.65) [527] | 1.58 (0.65) [343] | 1.63 (0.61) [148] | 1.62 (0.75) [36] |
| Neurofilament light chain, pg/mL  Visit 3 (1993-95) | 12.9 (7.6) [1501] | 12.7 (8.3) [998] | 12.8 (5.5) [351] | 14.2 (6.4) [152] |
| Visit 5 (2011-13) | 25.3 (13.5) [1499] | 25.1 (13.7) [997] | 24.4 (12.3) [350] | 28.6 (14.4) [152] |
| Visit 6 & 7 (2016-19) | 29.6 (16.5) [527] | 29.1 (15.7) [343] | 29.8 (18.0) [148] | 33.6 (17.4) [36] |
| Log2 neurofilament light chain  Visit 3 (1993-95) | 3.52 (0.67) [1501] | 3.49 (0.69) [998] | 3.56 (0.59) [351] | 3.68 (0.67) [152] |
| Visit 5 (2011-13) | 4.49 (0.70) [1499] | 4.47 (0.71) [997] | 4.45 (0.66) [350] | 4.68 (0.66) [152] |
| Visit 6 & 7 (2016-19) | 4.72 (0.66) [527] | 4.70 (0.66) [343] | 4.73 (0.66) [148] | 4.91 (0.66) [36] |
| Glial fibrillary acidic protein, pg/mL  Visit 3 (1993-95) | 106.3 (63.1) [1501] | 107.8 (69.6) [998] | 99.4 (46.1) [351] | 112.9 (49.4) [152] |
| Visit 5 (2011-13) | 195.4 (104.8) [1499] | 195.9 (101.6) [997] | 181.9 (108.7) [350] | 223.5 (111.1) [152] |
| Visit 6 & 7 (2016-19) | 208.2 (98.5) [527] | 210.4 (99.0) [343] | 197.5 (88.2) [148] | 230.5 (128.2) [36] |
| Log2 glial fibrillary acidic protein  Visit 3 (1993-95) | 6.57 (0.67) [1501] | 6.58 (0.69) [998] | 6.50 (0.63) [351] | 6.68 (0.65) [152] |
| Visit 5 (2011-13) | 7.44 (0.69) [1499] | 7.45 (0.69) [997] | 7.34 (0.67) [350] | 7.65 (0.67) [152] |
| Visit 6 & 7 (2016-19) | 7.56 (0.63) [527] | 7.57 (0.64) [343] | 7.50 (0.60) [148] | 7.69 (0.65) [36] |
| Dementia on or before 2020 | 391/1501 (26%) | 247/998 (25%) | 91/351 (26%) | 53/152 (35%) |
| Death on or before 2020 | 392/1501 (26%) | 262/998 (26%) | 79/351 (23%) | 51/152 (34%) |

Abbreviations: GED, General Educational Development credential; kg, kilogram; m^2^, meters squared; min, minute; mL, milliliters; pg, picograms.

^a^TBI before Visit 3 or on an unknown date.

**Data are n (%) or mean (SD**) [total]. **Denominators for percentages are based on the number of participants with complete data.**

# **eTable 10. Sensitivity Analysis of Covariate-Adjusted Change (95% CI) in Standardized Plasma Biomarkers by Traumatic Brain Injury (TBI) Status, the ARIC Study 1993-2019 (N=1,501)**

|  | **Difference at**  **Median Time of TBI** | **Change Per Decade** | **Difference** |
| --- | --- | --- | --- |
|  | **ß (95% CI)** | **ß (95% CI)** | **ß (95% CI)** |
|  | **Aß42/Aß40 Ratio^** | | |
| **Any Head Injury** |  |  |  |
| No TBI (N=998) | Referent | 0.043 (0.015, 0.071) | Referent |
| TBI (N=503) | 0.072 (0.021, 0.124) | 0.011 (-0.027, 0.049) | -0.032 (-0.072, 0.008) |
| **TBI Frequency** |  |  |  |
| No TBI (N=998) | Referent | 0.042 (0.015, 0.070) | Referent |
| 1 TBI (N=142) | 0.109 (0.038, 0.180) | 0.033 (-0.025, 0.092) | -0.009 (-0.069, 0.051) |
| 2+ TBIs (N=361) | 0.030 (-0.032, 0.092) | 0.007 (-0.037, 0.052) | -0.035 (-0.082, 0.012) |
| **TBI Severity^a^** |  |  |  |
| No TBI (N=998) | Referent | 0.035 (0.005, 0.065) | Referent |
| Mild TBI (N=137) | 0.068 (-0.063, 0.199) | -0.078 (-0.188, 0.032) | -0.113 (-0.224, -0.002) |
| Moderate, Severe, Penetrating TBI (N=41) | 0.208 (0.002, 0.414) | -0.037 (-0.205, 0.131) | -0.072 (-0.241, 0.097) |
|  | **Log2 p-Tau181** | | |
| **Any Head Injury** |  |  |  |
| No TBI (N=998) | Referent | 0.153 (0.109, 0.196) | Referent |
| TBI (N=503) | 0.059 (-0.033, 0.151) | 0.116 (0.057, 0.175) | -0.037 (-0.102, 0.029) |
| **TBI Frequency** |  |  |  |
| No TBI (N=998) | Referent | 0.156 (0.112, 0.199) | Referent |
| 1 TBI (N=142) | 0.004 (-0.127, 0.135) | 0.159 (0.057, 0.260) | 0.003 (-0.103, 0.109) |
| 2+ TBIs (N=361) | 0.060 (-0.071, 0.191) | 0.080 (0.011, 0.149) | -0.076 (-0.150, -0.001) |
| **TBI Severity^a^** |  |  |  |
| No TBI (N=998) | Referent | 0.152 (0.104, 0.200) | Referent |
| Mild TBI (N=137) | 0.113 (-0.098, 0.324) | -0.039 (-0.207, 0.129) | -0.192 (-0.362, -0.022) |
| Moderate, Severe, Penetrating TBI (N=41) | 0.338 (0.008, 0.668) | -0.114 (-0.363, 0.135) | -0.266 (-0.518, -0.015) |
|  | **Log2 NfL** | | |
| **Any Head Injury** |  |  |  |
| No TBI (N=998) | Referent | 0.242 (0.200, 0.284) | Referent |
| TBI (N=503) | 0.081 (-0.008, 0.170) | 0.224 (0.168, 0.280) | -0.018 (-0.081, 0.045) |
| **TBI Frequency** |  |  |  |
| No TBI (N=998) | Referent | 0.248 (0.206, 0.290) | Referent |
| 1 TBI (N=142) | -0.062 (-0.189, 0.065) | 0.262 (0.163, 0.360) | 0.014 (-0.089, 0.116) |
| 2+ TBIs (N=361) | 0.143 (0.014, 0.273) | 0.172 (0.106, 0.237) | -0.076 (-0.148, -0.005) |
| **TBI Severity^a^** |  |  |  |
| No TBI (N=998) | Referent | 0.235 (0.192, 0.278) | Referent |
| Mild TBI (N=137) | 0.079 (-0.113, 0.270) | 0.097 (-0.053, 0.246) | -0.138 (-0.290, 0.013) |
| Moderate, Severe, Penetrating TBI (N=41) | 0.371 (0.073, 0.670) | 0.194 (-0.026, 0.413) | -0.041 (-0.263, 0.181) |
|  | **Log2 GFAP** | | |
| **Any Head Injury** |  |  |  |
| No TBI (N=998) | Referent | 0.172 (0.133, 0.212) | Referent |
| TBI (N=503) | 0.086 (-0.005, 0.178) | 0.174 (0.121, 0.227) | 0.002 (-0.057, 0.060) |
| **TBI Frequency** |  |  |  |
| No TBI (N=998) | Referent | 0.179 (0.140, 0.219) | Referent |
| 1 TBI (N=142) | -0.009 (-0.129, 0.112) | 0.282 (0.189, 0.375) | 0.103 (0.006, 0.200) |
| 2+ TBIs (N=361) | 0.138 (0.011, 0.265) | 0.089 (0.028, 0.150) | -0.090 (-0.156, -0.024) |
| **TBI Severity^a^** |  |  |  |
| No TBI (N=998) | Referent | 0.176 (0.133, 0.219) | Referent |
| Mild TBI (N=137) | 0.098 (-0.100, 0.295) | -0.052 (-0.200, 0.096) | -0.228 (-0.378, -0.079) |
| Moderate, Severe, Penetrating TBI (N=41) | 0.316 (0.008, 0.623) | 0.042 (-0.173, 0.258) | -0.134 (-0.351, 0.084) |

Abbreviations: Aβ, amyloid-β; CI, confidence intervals; GFAP, glial fibrillary acidic protein; NfL, neurofilament light chain; p-tau181, phosphorylated Tau-181; TBI, traumatic brain injury.

^Indicates that the Aβ42/Aβ40 ratio was inverted so that higher values denote greater risk. For log2 p-tau181, log2 NfL, and log2 GFAP positive values indicate a faster rate of change and negative values indicate a slower rate.

^a^TBI severity data was only available in a subset of participants (N=1176).

The analytic sample was restricted to pre-dementia plasma biomarkers measured using the Quanterix Simoa platform. Parameter estimates generated from linear mixed effects models. First TBI defined as occurring 12.8 years after Visit 3 and second TBI defined as occurring 19.0 years after Visit 3 (median time of injuries). Biomarkers standardized to Visit 3 by mean-centring and dividing by 0.0459 for Aβ42/Aβ40 ratio, 0.8033 for log2 p-tau181, 0.6920 for log2 NfL, and 0.6867 for log2 GFAP. Models adjusted for age, sex, race-center, and education as time-invariant covariates and estimated glomerular filtration rate and body mass index as time-varying covariates. An interaction was specified between each time-invariant covariate and time. Multiple imputation by chained equations was employed to impute missing covariates. Inverse probability weighting was used to account for selection bias and informative attrition.

# **eTable 11. Characteristics of Primary Analysis Population of Association of Plasma Biomarkers and Incident Dementia Stratified by Incident Traumatic Brain Injury (TBI) Status (N=1,047)**

|  | **Overall** | **No TBI (N=909)** | **TBI (N=138)** |
| --- | --- | --- | --- |
| **Visit 5 (2011-13)** Age, years | 76.6 (5.3) [1047] | 76.4 (5.3) [909] | 77.8 (5.4) [138] |
| Female sex | 690/1047 (66%) | 592/909 (65%) | 98/138 (71%) |
| Race and center  Black, Jackson, Mississippi | 286/1047 (27%) | 265/909 (29%) | 21/138 (15%) |
| White, Washington County, Maryland | 279/1047 (27%) | 233/909 (26%) | 46/138 (33%) |
| White, Minneapolis suburbs, Minnesota | 243/1047 (23%) | 203/909 (22%) | 40/138 (29%) |
| White, Forsyth County, North Carolina | 222/1047 (21%) | 193/909 (21%) | 29/138 (21%) |
| Black, Forsyth County, North Carolina | 17/1047 (2%) | 15/909 (2%) | 2/138 (1%) |
| Education  Less than completed high school | 133/1047 (13%) | 117/909 (13%) | 16/138 (12%) |
| High school, GED, or vocational school | 437/1047 (42%) | 380/909 (42%) | 57/138 (41%) |
| Some college, graduate, or professional school | 477/1047 (46%) | 412/909 (45%) | 65/138 (47%) |
| One or more apolipoprotein E alleles | 267/998 (27%) | 234/868 (27%) | 33/130 (25%) |
| Cigarette use  Current | 54/1047 (5%) | 47/909 (5%) | 7/138 (5%) |
| Former | 511/1047 (49%) | 443/909 (49%) | 68/138 (49%) |
| Never | 482/1047 (46%) | 419/909 (46%) | 63/138 (46%) |
| Alcohol use  Current | 471/1020 (46%) | 404/887 (46%) | 67/133 (50%) |
| Former | 303/1020 (30%) | 268/887 (30%) | 35/133 (26%) |
| Never | 246/1020 (24%) | 215/887 (24%) | 31/133 (23%) |
| Diabetes | 326/1047 (31%) | 749/909 (82%) | 121/138 (88%) |
| Hypertension | 870/1047 (83%) | 404/909 (46%) | 67/138 (50%) |
| Body mass index, kg/m^2^ | 28.3 (5.2) [1043] | 28.3 (5.1) [905] | 28.6 (5.7) [138] |
| Estimated glomerular filtration rate, mL/min/1.73m^2^ | 70.4 (17.2) [1046] | 70.9 (17.2) [908] | 66.7 (16.9) [138] |
| **Plasma Assays** Amyloid-ß 42, pg/mL  Visit 3 (1993-95) | 3.76 (1.74) [1047] | 3.79 (1.74) [909] | 3.61 (1.71) [138] |
| Visit 5 (2011-13) | 6.34 (1.87) [1046] | 6.30 (1.87) [908] | 6.59 (1.86) [138] |
| Change per decade from Visit 3 to Visit 5 | 1.45 (1.18) [1046] | 1.42 (1.17) [908] | 1.67 (1.23) [138] |
| Amyloid-ß 40, pg/mL  Visit 3 (1993-95) | 58.9 (28.5) [1047] | 59.2 (28.3) [909] | 57.5 (29.8) [138] |
| Visit 5 (2011-13) | 108.0 (25.2) [1046] | 107.0 (25.0) [908] | 114.7 (25.2) [138] |
| Change per decade from Visit 3 to Visit 5 | 27.6 (18.5) [1046] | 27.0 (18.3) [908] | 32.0 (19.7) [138] |
| Aß42/Aß40 ratio  Visit 3 (1993-95) | 0.073 (0.046) [1047] | 0.073 (0.046) [909] | 0.074 (0.047) [138] |
| Visit 5 (2011-13) | 0.059 (0.014) [1046] | 0.060 (0.014) [908] | 0.058 (0.014) [138] |
| Change per decade from Visit 3 to Visit 5 | -0.008 (0.027) [1046] | -0.008 (0.027) [908] | -0.009 (0.026) [138] |
| Phosphorylated tau-181   Visit 3 (1993-95) | 1.56 (0.97) [1046] | 1.55 (0.95) [908] | 1.61 (1.09) [138] |
| Visit 5 (2011-13) | 3.11 (1.73) [1044] | 3.09 (1.75) [906] | 3.22 (1.61) [138] |
| Change per decade from Visit 3 to Visit 5 | 0.88 (0.91) [1043] | 0.87 (0.90) [905] | 0.91 (0.97) [138] |
| Log2 phosphorylated tau-181  Visit 3 (1993-95) | 0.42 (0.80) [1046] | 0.43 (0.78) [908] | 0.41 (0.95) [138] |
| Visit 5 (2011-13) | 1.47 (0.66) [1044] | 1.46 (0.66) [906] | 1.53 (0.69) [138] |
| Change per decade from Visit 3 to Visit 5 | 0.59 (0.45) [1043] | 0.59 (0.44) [905] | 0.63 (0.52) [138] |
| Neurofilament light chain, pg/mL  Visit 3 (1993-95) | 12.6 (7.9) [1047] | 12.4 (8.1) [909] | 14.0 (6.2) [138] |
| Visit 5 (2011-13) | 24.7 (13.2) [1046] | 24.3 (13.0) [908] | 27.7 (13.8) [138] |
| Change per decade from Visit 3 to Visit 5 | 6.8 (7.0) [1046] | 6.7 (7.0) [908] | 7.6 (6.9) [138] |
| Log2 neurofilament light chain  Visit 3 (1993-95) | 3.49 (0.68) [1047] | 3.46 (0.68) [909] | 3.67 (0.64) [138] |
| Visit 5 (2011-13) | 4.46 (0.70) [1046] | 4.43 (0.70) [908] | 4.64 (0.65) [138] |
| Change per decade from Visit 3 to Visit 5 | 0.55 (0.36) [1046] | 0.55 (0.36) [908] | 0.54 (0.35) [138] |
| Glial fibrillary acidic protein, pg/mL  Visit 3 (1993-95) | 107.0 (64.6) [1047] | 106.4 (66.6) [909] | 111.1 (49.0) [138] |
| Visit 5 (2011-13) | 196.1 (103.3) [1046] | 192.2 (101.2) [908] | 221.9 (113.2) [138] |
| Change per decade from Visit 3 to Visit 5 | 50.1 (49.3) [1046] | 48.3 (49.2) [908] | 61.9 (48.4) [138] |
| Log2 glial fibrillary acidic protein  Visit 3 (1993-95) | 6.57 (0.69) [1047] | 6.56 (0.69) [909] | 6.66 (0.65) [138] |
| Visit 5 (2011-13) | 7.45 (0.70) [1046] | 7.42 (0.69) [908] | 7.63 (0.68) [138] |
| Change per decade from Visit 3 to Visit 5 | 0.49 (0.32) [1046] | 0.49 (0.32) [908] | 0.54 (0.30) [138] |
| Dementia on or before 2020 | 237/1047 (23%) | 193/909 (21%) | 44/138 (32%) |
| Death on or before 2020 | 235/1047 (22%) | 197/909 (22%) | 38/138 (28%) |

Abbreviations: GED, General Educational Development credential; kg, kilogram; m^2^, meters squared; min, minute; mL, milliliters; pg, picograms.

**Data are n (%) or mean (SD**) [total]. **Denominators for percentages are based on the number of participants with complete data.**

# **eTable 12. Covariate-Adjusted Association of Standardized Plasma Biomarkers with Incident Dementia in Late-Life by Traumatic Brain Injury (TBI) Frequency, Severity, and Timing of TBI, the ARIC Study 2011-2020 (N=1,047)**

|  | **Aß42/Aß40 Ratio^** | | | **Log2 p-Tau181** | | | **Log2 NfL** | | | **Log2 GFAP** | | |
| --- | --- | --- | --- | --- | --- | --- | --- | --- | --- | --- | --- | --- |
|  | **HR (95% CI)** | **P for Interaction** | | **HR (95% CI)** | **P for Interaction** | | **HR (95% CI)** | **P for Interaction** | | **HR (95% CI)** | **P for Interaction** | |
|  |  | **Multiplicative** | **Additive** |  | **Multiplicative** | **Additive** |  | **Multiplicative** | **Additive** |  | **Multiplicative** | **Additive** |
|  | **Standardized^a^ Plasma Biomarkers Measured in Midlife (Visit 3, 1993-95)** | | | | | | | | | | | |
| **TBI Frequency** |  |  |  |  |  |  |  |  |  |  |  |  |
| No TBI (N=909) | 1.18 (1.02, 1.37) |  |  | 1.15 (1.05, 1.26) |  |  | 0.95 (0.86, 1.05) |  |  | 0.95 (0.86, 1.04) |  |  |
| 1 TBI (N=72) | 0.83 (0.65, 1.05) | 0.012 | 0.015 | 1.19 (0.95, 1.49) | 0.77 | 0.47 | 1.05 (0.80, 1.39) | 0.48 | 0.56 | 1.04 (0.78, 1.39) | 0.53 | 0.62 |
| 2+ TBIs (N=66) | 5.33 (2.00, 14.24) | 0.0027 | 0.072 | 1.18 (0.85, 1.63) | 0.88 | 0.85 | 0.93 (0.64, 1.36) | 0.94 | 0.93 | 1.02 (0.69, 1.52) | 0.71 | 0.73 |
| **TBI Severity^d^** |  |  |  |  |  |  |  |  |  |  |  |  |
| No TBI (N=909) | 1.17 (1.01, 1.36) |  |  | 1.15 (1.04, 1.26) |  |  | 0.96 (0.86, 1.06) |  |  | 0.97 (0.88, 1.06) |  |  |
| Mild TBI (N=93) | 0.81 (0.61, 1.09) | 0.028 | 0.037 | 1.10 (0.89, 1.36) | 0.73 | 0.85 | 1.07 (0.83, 1.37) | 0.42 | 0.50 | 1.02 (0.79, 1.31) | 0.69 | 0.78 |
| Moderate, Severe,  Penetrating TBI (N=32) | 2.28 (1.04, 5.00) | 0.096 | 0.21 | 1.30 (0.91, 1.85) | 0.49 | 0.45 | 0.85 (0.55, 1.30) | 0.58 | 0.56 | 0.62 (0.33, 1.16) | 0.17 | 0.11 |
| **Timing of TBI** |  |  |  |  |  |  |  |  |  |  |  |  |
| No TBI (N=909) | 1.18 (1.02, 1.37) |  |  | 1.16 (1.06, 1.28) |  |  | 0.95 (0.85, 1.05) |  |  | 0.96 (0.87, 1.05) |  |  |
| Before Visit 5 (N=99) | 0.97 (0.73, 1.28) | 0.22 | 0.26 | 1.19 (0.95, 1.49) | 0.85 | 0.68 | 1.10 (0.85, 1.42) | 0.29 | 0.31 | 1.07 (0.81, 1.42) | 0.44 | 0.47 |
| After Visit 5 (N=39) | 1.44 (0.69, 2.99) | 0.61 | 0.46 | 0.87 (0.66, 1.15) | 0.055 | 0.19 | 0.76 (0.43, 1.35) | 0.46 | 0.37 | 0.58 (0.38, 0.87) | 0.018 | 0.0088 |
|  | **Standardized^b^ Plasma Biomarkers Measured in Late-Life (Visit 5, 2011-13)** | | | | | | | | | | | |
| **TBI Frequency** |  |  |  |  |  |  |  |  |  |  |  |  |
| No TBI (N=909) | 1.38 (1.26, 1.50) |  |  | 1.54 (1.41, 1.69) |  |  | 1.90 (1.69, 2.13) |  |  | 1.40 (1.27, 1.54) |  |  |
| 1 TBI (N=72) | 1.23 (0.91, 1.67) | 0.49 | 0.93 | 2.57 (1.89, 3.50) | 0.0013 | 0.0004 | 2.70 (2.01, 3.63) | 0.017 | 0.0011 | 1.41 (1.10, 1.82) | 0.94 | 0.34 |
| 2+ TBIs (N=66) | 1.69 (1.36, 2.10) | 0.082 | 0.14 | 0.91 (0.65, 1.27) | 0.0020 | 0.0008 | 1.46 (1.05, 2.03) | 0.11 | 0.14 | 1.48 (1.04, 2.09) | 0.76 | 0.75 |
| **TBI Severity^d^** |  |  |  |  |  |  |  |  |  |  |  |  |
| No TBI (N=909) | 1.38 (1.27, 1.50) |  |  | 1.53 (1.40, 1.68) |  |  | 1.90 (1.69, 2.14) |  |  | 1.41 (1.28, 1.56) |  |  |
| Mild TBI (N=93) | 1.23 (1.00, 1.50) | 0.30 | 0.84 | 1.62 (1.26, 2.07) | 0.69 | 0.069 | 1.80 (1.42, 2.30) | 0.67 | 0.061 | 1.30 (1.02, 1.65) | 0.51 | 0.55 |
| Moderate, Severe,  Penetrating TBI (N=32) | 1.93 (1.39, 2.69) | 0.055 | 0.072 | 2.19 (1.38, 3.47) | 0.13 | 0.12 | 2.52 (1.45, 4.38) | 0.32 | 0.33 | 1.58 (1.02, 2.45) | 0.62 | 0.53 |
| **Timing of TBI** |  |  |  |  |  |  |  |  |  |  |  |  |
| No TBI (N=909) | 1.38 (1.26, 1.50) |  |  | 1.54 (1.40, 1.68) |  |  | 1.89 (1.68, 2.13) |  |  | 1.42 (1.28, 1.57) |  |  |
| Before Visit 5 (N=99) | 1.52 (1.24, 1.86) | 0.37 | 0.21 | 1.58 (1.22, 2.03) | 0.85 | 0.47 | 1.88 (1.45, 2.44) | 0.98 | 0.46 | 1.35 (1.05, 1.74) | 0.72 | 0.98 |
| After Visit 5 (N=39) | 1.48 (0.91, 2.40) | 0.77 | 0.31 | 1.94 (1.32, 2.84) | 0.24 | 0.014 | 5.03 (2.93, 8.64) | 0.0005 | 0.0001 | 1.31 (0.94, 1.82) | 0.63 | 0.37 |
|  | **Standardized^c^ Plasma Biomarkers Change Per Decade Between Midlife and Late-Life** | | | | | | | | | | | |
| **TBI Frequency** |  |  |  |  |  |  |  |  |  |  |  |  |
| No TBI (N=909) | 1.03 (0.94, 1.13) |  |  | 1.21 (1.12, 1.32) |  |  | 1.54 (1.41, 1.68) |  |  | 1.45 (1.33, 1.58) |  |  |
| 1 TBI (N=72) | 1.22 (0.93, 1.59) | 0.24 | 0.24 | 1.30 (1.04, 1.64) | 0.56 | 0.25 | 1.87 (1.40, 2.50) | 0.20 | 0.040 | 1.34 (1.04, 1.74) | 0.58 | 0.74 |
| 2+ TBIs (N=66) | 0.78 (0.49, 1.23) | 0.22 | 0.17 | 0.86 (0.64, 1.15) | 0.020 | 0.0099 | 1.32 (0.95, 1.83) | 0.35 | 0.41 | 1.45 (1.01, 2.09) | 0.98 | 0.98 |
| **TBI Severity^d^** |  |  |  |  |  |  |  |  |  |  |  |  |
| No TBI (N=909) | 1.03 (0.94, 1.13) |  |  | 1.22 (1.12, 1.33) |  |  | 1.55 (1.42, 1.69) |  |  | 1.43 (1.31, 1.57) |  |  |
| Mild TBI (N=93) | 1.31 (1.02, 1.68) | 0.081 | 0.090 | 1.16 (0.94, 1.42) | 0.62 | 0.73 | 1.34 (1.05, 1.71) | 0.26 | 0.75 | 1.25 (0.98, 1.60) | 0.30 | 0.88 |
| Moderate, Severe,  Penetrating TBI (N=32) | 0.63 (0.37, 1.09) | 0.076 | 0.050 | 1.05 (0.81, 1.35) | 0.25 | 0.29 | 2.51 (1.43, 4.42) | 0.096 | 0.099 | 2.67 (1.56, 4.59) | 0.026 | 0.033 |
| **Timing of TBI** |  |  |  |  |  |  |  |  |  |  |  |  |
| No TBI (N=909) | 1.02 (0.93, 1.12) |  |  | 1.20 (1.10, 1.31) |  |  | 1.55 (1.41, 1.69) |  |  | 1.45 (1.33, 1.58) |  |  |
| Before Visit 5 (N=99) | 1.21 (0.98, 1.49) | 0.16 | 0.16 | 1.04 (0.87, 1.25) | 0.16 | 0.26 | 1.30 (1.00, 1.68) | 0.20 | 0.45 | 1.19 (0.92, 1.53) | 0.14 | 0.24 |
| After Visit 5 (N=39) | 0.81 (0.43, 1.54) | 0.49 | 0.45 | 1.63 (1.20, 2.21) | 0.063 | 0.022 | 4.31 (2.67, 6.96) | <0.0001 | 0.0003 | 2.37 (1.63, 3.45) | 0.012 | 0.0027 |

Abbreviations: Aβ, amyloid-β; CI, confidence intervals; GFAP, glial fibrillary acidic protein; HR, hazard ratio; NfL, neurofilament light chain; p-tau181, phosphorylated Tau-181; TBI, traumatic brain injury.

^Indicates that the Aβ42/Aβ40 ratio was inverted so that higher values denote greater risk of incident dementia.

^a^Biomarkers standardized to Visit 3 by mean-centring and dividing by 0.0459 for Aβ42/Aβ40 ratio, 0.7997 for log2 p-tau181, 0.6824 for log2 NfL, and 0.6885 for log2 GFAP.

^b^Biomarkers standardized to Visit 5 by mean-centring and dividing by 0.0143 for Aβ42/Aβ40 ratio, 0.6631 for log2 p-tau181, 0.6990 for log2 NfL, and 0.6951 for log2 GFAP.

^c^Change in biomarkers per decade standardized by mean-centring and dividing by 0.0270 for Aβ42/Aβ40 ratio, 0.4506 for log2 p-tau181, 0.3589 for log2 NfL, and 0.3172 for log2 GFAP.

^d^TBI severity data was only available in a subset of participants (N=1034).

Multiplicative interactions show the HR (95% CI) and p-value for TBI x biomarker interaction term. Additive interactions show the exponentiated relative excess risk due to interaction (RERI) (95% CI) and p-values.

The analytic sample was restricted to pre-dementia plasma biomarkers measured using the Quanterix Simoa platform. Hazard ratios and 95% confidence intervals for incident dementia were calculated from cause-specific Cox proportional hazards regression models. Models adjusted for Visit 5 measures of age, sex, race-center, education, estimated glomerular filtration rate, and body mass index as time-invariant covariates. Multiple imputation by chained equations was employed to impute missing covariates. Inverse probability weighting was used to account for selection bias.

# **eTable 13. Covariate-Adjusted Association of Standardized Plasma Biomarkers with Incident Dementia in Late-Life by Self-Reported or ICD-9/10 Code Identified Traumatic Brain Injury (TBI) Status, the ARIC Study 2011-2020 (N=1,047)**

|  | **Aß42/Aß40 Ratio^** | | | **Log2 p-Tau181** | | | **Log2 NfL** | | | **Log2 GFAP** | | |
| --- | --- | --- | --- | --- | --- | --- | --- | --- | --- | --- | --- | --- |
|  | **HR (95% CI)** | **P for Interaction** | | **HR (95% CI)** | **P for Interaction** | | **HR (95% CI)** | **P for Interaction** | | **HR (95% CI)** | **P for Interaction** | |
|  |  | **Multiplicative** | **Additive** |  | **Multiplicative** | **Additive** |  | **Multiplicative** | **Additive** |  | **Multiplicative** | **Additive** |
|  | **Standardized^a^ Plasma Biomarkers Measured in Midlife (Visit 3, 1993-95)** | | | | | | | | | | | |
| **Self-Reported TBI** |  |  |  |  |  |  |  |  |  |  |  |  |
| No TBI (N=996) | 1.12 (0.99, 1.28) | 0.013 | 0.13 | 1.15 (1.06, 1.26) | 0.70 | 0.70 | 0.96 (0.87, 1.06) | 0.72 | 0.72 | 0.96 (0.88, 1.06) | 0.93 | 0.93 |
| TBI (N=51) | 4.93 (1.54, 15.78) |  |  | 1.23 (0.89, 1.71) |  |  | 1.04 (0.68, 1.58) |  |  | 0.94 (0.60, 1.48) |  |  |
| **ICD-9/10 Code**  **Identified TBI** |  |  |  |  |  |  |  |  |  |  |  |  |
| No TBI (N=922) | 1.18 (1.01, 1.37) | 0.59 | 0.92 | 1.15 (1.05, 1.27) | 0.85 | 0.64 | 0.95 (0.86, 1.05) | 0.49 | 0.60 | 0.97 (0.88, 1.06) | 0.65 | 0.54 |
| TBI (N=125) | 1.08 (0.82, 1.42) |  |  | 1.13 (0.96, 1.34) |  |  | 1.03 (0.83, 1.28) |  |  | 0.92 (0.73, 1.15) |  |  |
|  | **Standardized^b^ Plasma Biomarkers Measured in Late-Life (Visit 5, 2011-13)** | | | | | | | | | | | |
| **Self-Reported TBI** |  |  |  |  |  |  |  |  |  |  |  |  |
| No TBI (N=996) | 1.36 (1.26, 1.47) | 0.0049 | 0.023 | 1.55 (1.43, 1.69) | 0.74 | 0.98 | 1.93 (1.72, 2.17) | 0.43 | 0.45 | 1.42 (1.29, 1.56) | 0.96 | 0.94 |
| TBI (N=51) | 2.10 (1.57, 2.82) |  |  | 1.66 (1.12, 2.48) |  |  | 1.68 (1.18, 2.38) |  |  | 1.43 (0.98, 2.09) |  |  |
| **ICD-9/10 Code**  **Identified TBI** |  |  |  |  |  |  |  |  |  |  |  |  |
| No TBI (N=922) | 1.38 (1.27, 1.51) | 0.84 | 0.26 | 1.54 (1.41, 1.68) | 0.37 | 0.016 | 1.89 (1.68, 2.13) | 0.72 | 0.0097 | 1.41 (1.28, 1.56) | 0.73 | 0.33 |
| TBI (N=125) | 1.35 (1.13, 1.62) |  |  | 1.70 (1.37, 2.11) |  |  | 1.97 (1.58, 2.46) |  |  | 1.36 (1.10, 1.67) |  |  |
|  | **Standardized^c^ Plasma Biomarkers Change Per Decade Between Midlife and Late-Life** | | | | | | | | | | | |
| **Self-Reported TBI** |  |  |  |  |  |  |  |  |  |  |  |  |
| No TBI (N=996) | 1.04 (0.96, 1.14) | 0.45 | 0.40 | 1.21 (1.12, 1.31) | 0.23 | 0.21 | 1.56 (1.44, 1.70) | 0.30 | 0.31 | 1.44 (1.33, 1.57) | 0.67 | 0.78 |
| TBI (N=51) | 0.82 (0.43, 1.55) |  |  | 1.03 (0.80, 1.33) |  |  | 1.29 (0.90, 1.85) |  |  | 1.59 (1.03, 2.45) |  |  |
| **ICD-9/10 Code**  **Identified TBI** |  |  |  |  |  |  |  |  |  |  |  |  |
| No TBI (N=922) | 1.03 (0.93, 1.13) | 0.69 | 0.61 | 1.21 (1.11, 1.32) | 0.24 | 0.84 | 1.55 (1.42, 1.70) | 0.84 | 0.19 | 1.43 (1.31, 1.56) | 0.85 | 0.16 |
| TBI (N=125) | 1.08 (0.87, 1.33) |  |  | 1.10 (0.95, 1.27) |  |  | 1.52 (1.22, 1.88) |  |  | 1.46 (1.18, 1.81) |  |  |

Abbreviations: Aβ, amyloid-β; CI, confidence intervals; GFAP, glial fibrillary acidic protein; HR, hazard ratio; ICD, international classification of diseases; NfL, neurofilament light chain; p-tau181, phosphorylated Tau-181; TBI, traumatic brain injury.

^Indicates that the Aβ42/Aβ40 ratio was inverted so that higher values denote greater risk of incident dementia.

^a^Biomarkers standardized to Visit 3 by mean-centring and dividing by 0.0459 for Aβ42/Aβ40 ratio, 0.7997 for log2 p-tau181, 0.6824 for log2 NfL, and 0.6885 for log2 GFAP.

^b^Biomarkers standardized to Visit 5 by mean-centring and dividing by 0.0143 for Aβ42/Aβ40 ratio, 0.6631 for log2 p-tau181, 0.6990 for log2 NfL, and 0.6951 for log2 GFAP.

^c^Change in biomarkers per decade standardized by mean-centring and dividing by 0.0270 for Aβ42/Aβ40 ratio, 0.4506 for log2 p-tau181, 0.3589 for log2 NfL, and 0.3172 for log2 GFAP.

Multiplicative interactions show the HR (95% CI) and p-value for TBI x biomarker interaction term. Additive interactions show the exponentiated relative excess risk due to interaction (RERI) (95% CI) and p-values.

The analytic sample was restricted to pre-dementia plasma biomarkers measured using the Quanterix Simoa platform. Hazard ratios and 95% confidence intervals for incident dementia were calculated from cause-specific Cox proportional hazards regression models. Models adjusted for Visit 5 measures of age, sex, race-center, education, estimated glomerular filtration rate, and body mass index as time-invariant covariates. Multiple imputation by chained equations was employed to impute missing covariates. Inverse probability weighting was used to account for selection bias.

# **eTable 14. Characteristics of Sensitivity Analysis Population of Association of Plasma Biomarkers and Incident Dementia Stratified by Timing of Incident Traumatic Brain Injury (TBI) Status (N=1,375)**

|  | **Overall** | **No TBI (N=909)** | **TBI Before Visit 5 (N=427)** | **TBI After Visit 5 (N=39)** |
| --- | --- | --- | --- | --- |
| **Visit 5 (2011-13)** Age, years | 76.5 (5.2) [1375] | 76.4 (5.3) [909] | 76.7 (5.2) [427] | 77.4 (5.5) [39] |
| Female sex | 834/1375 (61%) | 592/909 (65%) | 215/427 (50%) | 27/39 (69%) |
| Race and center  White, Washington County, Maryland | 367/1375 (27%) | 233/909 (26%) | 116/427 (27%) | 18/39 (46%) |
| White, Minneapolis suburbs, Minnesota | 344/1375 (25%) | 203/909 (22%) | 131/427 (31%) | 10/39 (26%) |
| Black, Jackson, Mississippi | 331/1375 (24%) | 265/909 (29%) | 62/427 (15%) | 4/39 (10%) |
| White, Forsyth County, North Carolina | 312/1375 (23%) | 193/909 (21%) | 112/427 (26%) | 7/39 (18%) |
| Black, Forsyth County, North Carolina | 21/1375 (2%) | 15/909 (2%) | 6/427 (1%) | 0/39 (0%) |
| Education  Less than completed high school | 166/1374 (12%) | 117/909 (13%) | 46/426 (11%) | 3/39 (8%) |
| High school, GED, or vocational school | 576/1374 (42%) | 380/909 (42%) | 176/426 (41%) | 20/39 (51%) |
| Some college, graduate, or professional school | 632/1374 (46%) | 412/909 (45%) | 204/426 (48%) | 16/39 (41%) |
| One or more apolipoprotein E alleles | 348/1309 (27%) | 234/868 (27%) | 104/405 (26%) | 10/36 (28%) |
| Cigarette use  Current | 67/1375 (5%) | 47/909 (5%) | 18/427 (4%) | 2/39 (5%) |
| Former | 701/1375 (51%) | 443/909 (49%) | 235/427 (55%) | 23/39 (59%) |
| Never | 607/1375 (44%) | 419/909 (46%) | 174/427 (41%) | 14/39 (36%) |
| Alcohol use  Current | 661/1337 (49%) | 404/887 (46%) | 235/412 (57%) | 22/38 (58%) |
| Former | 381/1337 (29%) | 268/887 (30%) | 104/412 (25%) | 9/38 (24%) |
| Never | 295/1337 (22%) | 215/887 (24%) | 73/412 (18%) | 7/38 (18%) |
| Diabetes | 426/1375 (31%) | 749/909 (82%) | 135/427 (32%) | 19/39 (49%) |
| Hypertension | 1109/1375 (81%) | 404/909 (46%) | 329/427 (77%) | 31/39 (80%) |
| Body mass index, kg/m^2^ | 28.4 (5.2) [1368] | 28.3 (5.1) [905] | 28.4 (5.1) [424] | 29.5 (5.9) [39] |
| Estimated glomerular filtration rate, mL/min/1.73m^2^ | 71.0 (17.4) [1374] | 70.9 (17.2) [908] | 71.1 (17.9) [427] | 69.6 (15.7) [39] |
| **Plasma Assays** Amyloid-ß 42, pg/mL  Visit 3 (1993-95) | 3.80 (1.71) [1375] | 3.79 (1.74) [909] | 3.85 (1.65) [427] | 3.56 (1.86) [39] |
| Visit 5 (2011-13) | 6.38 (1.84) [1373] | 6.30 (1.87) [908] | 6.51 (1.77) [426] | 6.84 (1.85) [39] |
| Change per decade from Visit 3 to Visit 5 | 1.45 (1.19) [1373] | 1.42 (1.17) [908] | 1.49 (1.22) [426] | 1.84 (1.30) [39] |
| Amyloid-ß 40, pg/mL  Visit 3 (1993-95) | 59.6 (28.1) [1375] | 59.2 (28.3) [909] | 60.5 (27.4) [427] | 59.2 (32.4) [39] |
| Visit 5 (2011-13) | 108.2 (25.0) [1373] | 107.0 (25.0) [908] | 109.9 (24.4) [426] | 116.5 (27.4) [39] |
| Change per decade from Visit 3 to Visit 5 | 27.4 (18.6) [1373] | 27.0 (18.3) [908] | 27.8 (19.0) [426] | 32.3 (21.6) [39] |
| Aß42/Aß40 ratio  Visit 3 (1993-95) | 0.073 (0.049) [1375] | 0.073 (0.046) [909] | 0.073 (0.056) [427] | 0.073 (0.049) [39] |
| Visit 5 (2011-13) | 0.060 (0.014) [1373] | 0.060 (0.014) [908] | 0.060 (0.012) [426] | 0.059 (0.012) [39] |
| Change per decade from Visit 3 to Visit 5 | -0.008 (0.029) [1373] | -0.008 (0.027) [908] | -0.008 (0.033) [426] | -0.008 (0.024) [39] |
| Phosphorylated tau-181   Visit 3 (1993-95) | 1.58 (0.99) [1374] | 1.55 (0.95) [908] | 1.64 (1.01) [427] | 1.74 (1.47) [39] |
| Visit 5 (2011-13) | 3.09 (1.74) [1372] | 3.09 (1.75) [906] | 3.07 (1.67) [427] | 3.42 (2.08) [39] |
| Change per decade from Visit 3 to Visit 5 | 0.85 (0.90) [1371] | 0.87 (0.90) [905] | 0.80 (0.87) [427] | 0.96 (1.33) [39] |
| Log2 phosphorylated tau-181  Visit 3 (1993-95) | 0.45 (0.79) [1374] | 0.43 (0.78) [908] | 0.51 (0.80) [427] | 0.44 (1.07) [39] |
| Visit 5 (2011-13) | 1.46 (0.67) [1372] | 1.46 (0.66) [906] | 1.46 (0.67) [427] | 1.54 (0.87) [39] |
| Change per decade from Visit 3 to Visit 5 | 0.57 (0.45) [1371] | 0.59 (0.44) [905] | 0.53 (0.46) [427] | 0.62 (0.56) [39] |
| Neurofilament light chain, pg/mL  Visit 3 (1993-95) | 12.6 (7.3) [1375] | 12.4 (8.1) [909] | 13.0 (5.8) [427] | 13.3 (4.6) [39] |
| Visit 5 (2011-13) | 24.4 (12.8) [1373] | 24.3 (13.0) [908] | 24.7 (12.5) [426] | 25.3 (11.3) [39] |
| Change per decade from Visit 3 to Visit 5 | 6.7 (6.7) [1373] | 6.7 (7.0) [908] | 6.6 (5.9) [426] | 6.8 (6.1) [39] |
| Log2 neurofilament light chain  Visit 3 (1993-95) | 3.50 (0.66) [1375] | 3.46 (0.68) [909] | 3.58 (0.61) [427] | 3.65 (0.51) [39] |
| Visit 5 (2011-13) | 4.44 (0.69) [1373] | 4.43 (0.70) [908] | 4.47 (0.65) [426] | 4.53 (0.62) [39] |
| Change per decade from Visit 3 to Visit 5 | 0.53 (0.35) [1373] | 0.55 (0.36) [908] | 0.51 (0.32) [426] | 0.50 (0.35) [39] |
| Glial fibrillary acidic protein, pg/mL  Visit 3 (1993-95) | 105.2 (60.9) [1375] | 106.4 (66.6) [909] | 102.0 (48.0) [427] | 111.6 (44.8) [39] |
| Visit 5 (2011-13) | 191.2 (103.0) [1373] | 192.2 (101.2) [908] | 186.6 (105.2) [426] | 218.8 (118.2) [39] |
| Change per decade from Visit 3 to Visit 5 | 48.4 (48.5) [1373] | 48.3 (49.2) [908] | 47.5 (46.6) [426] | 60.6 (53.2) [39] |
| Log2 glial fibrillary acidic protein  Visit 3 (1993-95) | 6.55 (0.68) [1375] | 6.56 (0.69) [909] | 6.53 (0.65) [427] | 6.68 (0.61) [39] |
| Visit 5 (2011-13) | 7.41 (0.69) [1373] | 7.42 (0.69) [908] | 7.38 (0.67) [426] | 7.59 (0.74) [39] |
| Change per decade from Visit 3 to Visit 5 | 0.48 (0.31) [1373] | 0.49 (0.32) [908] | 0.48 (0.29) [426] | 0.51 (0.29) [39] |
| Dementia on or before 2020 | 315/1375 (23%) | 193/909 (21%) | 106/427 (25%) | 16/39 (41%) |
| Death on or before 2020 | 295/1375 (22%) | 197/909 (22%) | 86/427 (20%) | 12/39 (31%) |

Abbreviations: GED, General Educational Development credential; kg, kilogram; m^2^, meters squared; min, minute; mL, milliliters; pg, picograms.

**Data are n (%) or mean (SD**) [total]. **Denominators for percentages are based on the number of participants with complete data.**

# **eTable 15. Sensitivity Analysis of Covariate-Adjusted Association of Standardized Plasma Biomarkers with Incident Dementia in Late-Life by Traumatic Brain Injury (TBI) Status, the ARIC Study 2011-2020 (N=1,375)**

|  | **Aß42/Aß40 Ratio^** | | | **Log2 p-Tau181** | | | **Log2 NfL** | | | **Log2 GFAP** | | |
| --- | --- | --- | --- | --- | --- | --- | --- | --- | --- | --- | --- | --- |
|  | **HR (95% CI)** | **P for Interaction** | | **HR (95% CI)** | **P for Interaction** | | **HR (95% CI)** | **P for Interaction** | | **HR (95% CI)** | **P for Interaction** | |
|  |  | **Multiplicative** | **Additive** |  | **Multiplicative** | **Additive** |  | **Multiplicative** | **Additive** |  | **Multiplicative** | **Additive** |
|  | **Standardized^a^ Plasma Biomarkers Measured in Midlife (Visit 3, 1993-95)** | | | | | | | | | | | |
| **TBI Frequency** |  |  |  |  |  |  |  |  |  |  |  |  |
| No TBI (N=986) | 1.14 (0.99, 1.30) |  |  | 1.15 (1.05, 1.25) |  |  | 0.93 (0.84, 1.02) |  |  | 0.98 (0.89, 1.07) |  |  |
| 1 TBI (N=90) | 0.80 (0.63, 1.02) | 0.011 | 0.0084 | 1.35 (1.10, 1.65) | 0.14 | 0.094 | 1.39 (1.11, 1.74) | 0.0005 | 0.0022 | 1.08 (0.86, 1.37) | 0.37 | 0.40 |
| 2+ TBIs (N=299) | 2.56 (1.57, 4.18) | 0.0010 | 0.017 | 1.14 (0.92, 1.42) | 0.95 | 0.98 | 0.73 (0.59, 0.91) | 0.034 | 0.031 | 1.09 (0.87, 1.37) | 0.34 | 0.35 |
| **TBI Severity^d^** |  |  |  |  |  |  |  |  |  |  |  |  |
| No TBI (N=986) | 1.17 (1.02, 1.33) |  |  | 1.19 (1.10, 1.29) |  |  | 0.94 (0.86, 1.03) |  |  | 1.01 (0.93, 1.09) |  |  |
| Mild TBI (N=105) | 0.82 (0.61, 1.12) | 0.040 | 0.054 | 1.10 (0.89, 1.37) | 0.51 | 0.96 | 1.15 (0.91, 1.46) | 0.11 | 0.16 | 1.17 (0.91, 1.49) | 0.25 | 0.23 |
| Moderate, Severe,  Penetrating TBI (N=32) | 2.13 (1.03, 4.39) | 0.10 | 0.20 | 1.30 (0.91, 1.86) | 0.63 | 0.52 | 0.85 (0.56, 1.29) | 0.64 | 0.59 | 0.64 (0.35, 1.20) | 0.16 | 0.10 |
| **Timing of TBI** |  |  |  |  |  |  |  |  |  |  |  |  |
| No TBI (N=986) | 1.17 (1.03, 1.34) |  |  | 1.19 (1.10, 1.29) |  |  | 0.95 (0.87, 1.04) |  |  | 1.02 (0.94, 1.10) |  |  |
| Before Visit 5 (N=350) | 0.99 (0.74, 1.33) | 0.31 | 0.38 | 1.29 (1.03, 1.61) | 0.52 | 0.37 | 1.02 (0.80, 1.30) | 0.58 | 0.62 | 0.99 (0.76, 1.30) | 0.87 | 0.89 |
| After Visit 5 (N=39) | 1.50 (0.71, 3.19) | 0.52 | 0.41 | 0.88 (0.67, 1.15) | 0.035 | 0.17 | 0.77 (0.43, 1.37) | 0.48 | 0.39 | 0.59 (0.39, 0.88) | 0.0086 | 0.0071 |
|  | **Standardized^b^ Plasma Biomarkers Measured in Late-Life (Visit 5, 2011-13)** | | | | | | | | | | | |
| **TBI Frequency** |  |  |  |  |  |  |  |  |  |  |  |  |
| No TBI (N=986) | 1.41 (1.30, 1.52) |  |  | 1.58 (1.45, 1.71) |  |  | 1.85 (1.66, 2.05) |  |  | 1.44 (1.31, 1.58) |  |  |
| 1 TBI (N=90) | 1.22 (0.99, 1.50) | 0.17 | 0.42 | 2.86 (2.29, 3.57) | <0.0001 | <0.0001 | 2.47 (1.97, 3.10) | 0.0087 | 0.0054 | 1.78 (1.46, 2.16) | 0.029 | 0.011 |
| 2+ TBIs (N=299) | 1.73 (1.47, 2.04) | 0.019 | 0.024 | 1.05 (0.85, 1.28) | 0.0001 | <0.0001 | 1.52 (1.23, 1.87) | 0.062 | 0.12 | 1.40 (1.16, 1.69) | 0.78 | 0.89 |
| **TBI Severity^d^** |  |  |  |  |  |  |  |  |  |  |  |  |
| No TBI (N=986) | 1.42 (1.32, 1.53) |  |  | 1.56 (1.44, 1.68) |  |  | 1.82 (1.65, 2.01) |  |  | 1.45 (1.33, 1.59) |  |  |
| Mild TBI (N=105) | 1.36 (1.12, 1.65) | 0.67 | 0.36 | 1.84 (1.46, 2.33) | 0.17 | 0.0071 | 2.10 (1.67, 2.63) | 0.23 | 0.0019 | 1.63 (1.32, 2.02) | 0.31 | 0.017 |
| Moderate, Severe,  Penetrating TBI (N=32) | 1.85 (1.34, 2.56) | 0.12 | 0.11 | 2.23 (1.42, 3.50) | 0.12 | 0.085 | 2.53 (1.45, 4.40) | 0.25 | 0.20 | 1.61 (1.04, 2.49) | 0.64 | 0.50 |
| **Timing of TBI** |  |  |  |  |  |  |  |  |  |  |  |  |
| No TBI (N=986) | 1.45 (1.35, 1.56) |  |  | 1.56 (1.44, 1.68) |  |  | 1.85 (1.68, 2.05) |  |  | 1.50 (1.37, 1.63) |  |  |
| Before Visit 5 (N=350) | 1.38 (1.13, 1.69) | 0.66 | 0.98 | 1.78 (1.41, 2.25) | 0.28 | 0.099 | 1.77 (1.39, 2.26) | 0.72 | 0.61 | 1.27 (1.01, 1.61) | 0.20 | 0.38 |
| After Visit 5 (N=39) | 1.51 (0.94, 2.41) | 0.87 | 0.29 | 2.00 (1.37, 2.91) | 0.20 | 0.0099 | 5.23 (3.04, 9.01) | 0.0002 | 0.0001 | 1.35 (0.96, 1.88) | 0.55 | 0.34 |
|  | **Standardized^c^ Plasma Biomarkers Change Per Decade Between Midlife and Late-Life** | | | | | | | | | | | |
| **TBI Frequency** |  |  |  |  |  |  |  |  |  |  |  |  |
| No TBI (N=986) | 1.05 (0.97, 1.13) |  |  | 1.24 (1.15, 1.34) |  |  | 1.57 (1.44, 1.71) |  |  | 1.41 (1.30, 1.54) |  |  |
| 1 TBI (N=90) | 1.26 (0.99, 1.60) | 0.14 | 0.16 | 1.47 (1.20, 1.80) | 0.10 | 0.076 | 1.42 (1.13, 1.80) | 0.41 | 0.87 | 1.74 (1.41, 2.15) | 0.054 | 0.033 |
| 2+ TBIs (N=299) | 0.92 (0.69, 1.21) | 0.34 | 0.31 | 0.93 (0.76, 1.14) | 0.0062 | 0.0024 | 1.69 (1.37, 2.09) | 0.49 | 0.44 | 1.22 (1.00, 1.49) | 0.17 | 0.21 |
| **TBI Severity^d^** |  |  |  |  |  |  |  |  |  |  |  |  |
| No TBI (N=986) | 1.04 (0.97, 1.13) |  |  | 1.21 (1.13, 1.31) |  |  | 1.55 (1.43, 1.67) |  |  | 1.40 (1.29, 1.51) |  |  |
| Mild TBI (N=105) | 1.36 (1.08, 1.72) | 0.035 | 0.045 | 1.28 (1.04, 1.56) | 0.64 | 0.22 | 1.52 (1.20, 1.93) | 0.88 | 0.23 | 1.48 (1.17, 1.86) | 0.64 | 0.13 |
| Moderate, Severe,  Penetrating TBI (N=32) | 0.63 (0.38, 1.05) | 0.049 | 0.032 | 1.05 (0.81, 1.37) | 0.31 | 0.38 | 2.29 (1.31, 4.00) | 0.17 | 0.13 | 2.50 (1.44, 4.33) | 0.039 | 0.035 |
| **Timing of TBI** |  |  |  |  |  |  |  |  |  |  |  |  |
| No TBI (N=986) | 1.04 (0.97, 1.13) |  |  | 1.20 (1.11, 1.30) |  |  | 1.56 (1.44, 1.69) |  |  | 1.43 (1.32, 1.54) |  |  |
| Before Visit 5 (N=350) | 1.16 (0.92, 1.45) | 0.39 | 0.37 | 1.09 (0.91, 1.30) | 0.31 | 0.50 | 1.32 (1.02, 1.69) | 0.20 | 0.48 | 1.16 (0.92, 1.48) | 0.11 | 0.21 |
| After Visit 5 (N=39) | 0.79 (0.42, 1.52) | 0.41 | 0.39 | 1.62 (1.20, 2.18) | 0.060 | 0.020 | 4.25 (2.65, 6.82) | <0.0001 | 0.0002 | 2.49 (1.71, 3.63) | 0.0046 | 0.0016 |

Abbreviations: Aβ, amyloid-β; CI, confidence intervals; GFAP, glial fibrillary acidic protein; HR, hazard ratio; NfL, neurofilament light chain; p-tau181, phosphorylated Tau-181; TBI, traumatic brain injury.

^Indicates that the Aβ42/Aβ40 ratio was inverted so that higher values denote greater risk of incident dementia.

^a^Biomarkers standardized to Visit 3 by mean-centring and dividing by 0.0459 for Aβ42/Aβ40 ratio, 0.7997 for log2 p-tau181, 0.6824 for log2 NfL, and 0.6885 for log2 GFAP.

^b^Biomarkers standardized to Visit 5 by mean-centring and dividing by 0.0143 for Aβ42/Aβ40 ratio, 0.6631 for log2 p-tau181, 0.6990 for log2 NfL, and 0.6951 for log2 GFAP.

^c^Change in biomarkers per decade standardized by mean-centring and dividing by 0.0270 for Aβ42/Aβ40 ratio, 0.4506 for log2 p-tau181, 0.3589 for log2 NfL, and 0.3172 for log2 GFAP.

^d^TBI severity data was only available in a subset of participants (N=1123).

Multiplicative interactions show the HR (95% CI) and p-value for TBI x biomarker interaction term. Additive interactions show the exponentiated relative excess risk due to interaction (RERI) (95% CI) and p-values.

The analytic sample was restricted to pre-dementia plasma biomarkers measured using the Quanterix Simoa platform. Hazard ratios and 95% confidence intervals for incident dementia were calculated from cause-specific Cox proportional hazards regression models. Models adjusted for Visit 5 measures of age, sex, race-center, education, estimated glomerular filtration rate, and body mass index as time-invariant covariates. Multiple imputation by chained equations was employed to impute missing covariates. Inverse probability weighting was used to account for selection bias.

# **eFigure 1.** **Study Timeline and Participant Follow-up Patterns, the ARIC Study 1990-2019**

# **eFigure 2. Covariate-Adjusted, Model-Based Estimates of Change in Plasma Biomarkers Over Time by Traumatic Brain Injury (TBI) Frequency, the ARIC Study 1993-2019 (N=1,150)**

Abbreviations: Aβ, amyloid-β; GFAP, glial fibrillary acidic protein; NfL, neurofilament light chain; p-tau181, phosphorylated Tau-181; TBI, traumatic brain injury.

The analytic sample was restricted to pre-dementia plasma biomarkers measured using the Quanterix Simoa platform. First TBI defined as occurring 12.8 years after Visit 3 and second TBI defined as occurring 19.0 years after Visit 3 (median time of injuries). Parameter estimates generated from linear mixed effects models that adjusted for age, sex, race-center, and education as time-invariant covariates and estimated glomerular filtration rate and body mass index as time-varying covariates. An interaction was specified between each time-invariant covariate and time. Multiple imputation by chained equations was employed to impute missing covariates. Inverse probability weighting was used to account for selection bias and informative attrition. P-tau181, NfL, and GFAP were base 2 log transformed prior to analysis and then converted back to their original scale for the visualization.

# **eFigure 3. Covariate-Adjusted, Model-Based Estimates of Change in Plasma Biomarkers Over Time by Traumatic Brain Injury (TBI) Severity, the ARIC Study 1993-2019 (N=1,131)**

Abbreviations: Aβ, amyloid-β; GFAP, glial fibrillary acidic protein; NfL, neurofilament light chain; p-tau181, phosphorylated Tau-181; TBI, traumatic brain injury.

The analytic sample was restricted to pre-dementia plasma biomarkers measured using the Quanterix Simoa platform. TBI defined as occurring 12.8 years after Visit 3 (median time of injury). Parameter estimates generated from linear mixed effects models that adjusted for age, sex, race-center, and education as time-invariant covariates and estimated glomerular filtration rate and body mass index as time-varying covariates. An interaction was specified between each time-invariant covariate and time. Multiple imputation by chained equations was employed to impute missing covariates. Inverse probability weighting was used to account for selection bias and informative attrition. P-tau181, NfL, and GFAP were base 2 log transformed prior to analysis and then converted back to their original scale for the visualization.

# **eFigure 4. Covariate-Adjusted, Model-Based Estimates of Change in Plasma Biomarkers Over Time by Self-Reported Traumatic Brain Injury (TBI) Status, the ARIC Study 1993-2019 (N=1,150)**

Abbreviations: Aβ, amyloid-β; GFAP, glial fibrillary acidic protein; NfL, neurofilament light chain; p-tau181, phosphorylated Tau-181; TBI, traumatic brain injury.

The analytic sample was restricted to pre-dementia plasma biomarkers measured using the Quanterix Simoa platform. TBI defined as occurring 12.8 years after Visit 3 (median time of injury). Parameter estimates generated from linear mixed effects models that adjusted for age, sex, race-center, and education as time-invariant covariates and estimated glomerular filtration rate and body mass index as time-varying covariates. An interaction was specified between each time-invariant covariate and time. Multiple imputation by chained equations was employed to impute missing covariates. Inverse probability weighting was used to account for selection bias and informative attrition. P-tau181, NfL, and GFAP were base 2 log transformed prior to analysis and then converted back to their original scale for the visualization.

# **eFigure 5. Covariate-Adjusted, Model-Based Estimates of Change in Plasma Biomarkers Over Time by ICD-9/10 Code Identified Traumatic Brain Injury (TBI) Status, the ARIC Study 1993-2019 (N=1,150)**

Abbreviations: Aβ, amyloid-β; GFAP, glial fibrillary acidic protein; NfL, neurofilament light chain; p-tau181, phosphorylated Tau-181; TBI, traumatic brain injury.

The analytic sample was restricted to pre-dementia plasma biomarkers measured using the Quanterix Simoa platform. TBI defined as occurring 12.8 years after Visit 3 (median time of injury). Parameter estimates generated from linear mixed effects models that adjusted for age, sex, race-center, and education as time-invariant covariates and estimated glomerular filtration rate and body mass index as time-varying covariates. An interaction was specified between each time-invariant covariate and time. Multiple imputation by chained equations was employed to impute missing covariates. Inverse probability weighting was used to account for selection bias and informative attrition. P-tau181, NfL, and GFAP were base 2 log transformed prior to analysis and then converted back to their original scale for the visualization.

# **eFigure 6. Sensitivity Analysis of Covariate-Adjusted Change (95% CI) in Standardized Plasma Biomarkers by Traumatic Brain Injury (TBI) Status, Excluding Biomarker Observations Occurring within 1-Year Post-TBI, the ARIC Study 1993-2019 (N=1,150)**

Abbreviations: Aβ, amyloid-β; CI, confidence intervals; GFAP, glial fibrillary acidic protein; NfL, neurofilament light chain; p-tau181, phosphorylated Tau-181; TBI, traumatic brain injury.

^Indicates that the Aβ42/Aβ40 ratio was inverted so that higher values denote greater risk.

^a^TBI severity data was only available in a subset of participants (N=1131).

The analytic sample was restricted to pre-dementia plasma biomarkers measured using the Quanterix Simoa platform. TBIs that occurred one year before plasma was obtained were censored. Parameter estimates generated from linear mixed effects models. First TBI defined as occurring 12.8 years after Visit 3 and second TBI defined as occurring 19.0 years after Visit 3 (median injury times). Biomarkers standardized to Visit 3 by mean-centering and dividing by 0.0459 for Aβ42/Aβ40 ratio, 0.8033 for log2 p-tau181, 0.6920 for log2 NfL, and 0.6867 for log2 GFAP. Models adjusted for age, sex, race-center, and education as time-invariant covariates and estimated glomerular filtration rate and body mass index as time-varying covariates. An interaction was specified between each time-invariant covariate and time. Multiple imputation by chained equations was employed to impute missing covariates. Inverse probability weighting was used to account for selection bias and informative attrition.

# **eFigure 7. Sensitivity Analysis of Covariate-Adjusted Association of Standardized Plasma Biomarkers with Incident Dementia in Late-Life by Any Traumatic Brain Injury (TBI) Status, Excluding Biomarker Observations Occurring within 1-Year Post-TBI, the ARIC Study 2011-2020 (N=1,047)**

Abbreviations: Aβ, amyloid-β; CI, confidence intervals; GFAP, glial fibrillary acidic protein; HR, hazard ratio; NfL, neurofilament light chain; p-tau181, phosphorylated Tau-181; TBI, traumatic brain injury.

^Aβ42/Aβ40 ratio inverted so that higher values denote greater risk of incident dementia.

^a^Biomarkers standardized to Visit 3 by mean-centering and dividing by 0.0459 for Aβ42/Aβ40 ratio, 0.7997 for log2 p-tau181, 0.6824 for log2 NfL, and 0.6885 for log2 GFAP.

^b^Biomarkers standardized to Visit 5 by mean-centering and dividing by 0.0143 for Aβ42/Aβ40 ratio, 0.6631 for log2 p-tau181, 0.6990 for log2 NfL, and 0.6951 for log2 GFAP.

^c^Change in biomarkers per decade standardized by mean-centering and dividing by 0.0270 for Aβ42/Aβ40 ratio, 0.4506 for log2 p-tau181, 0.3589 for log2 NfL, and 0.3172 for log2 GFAP.

Multiplicative interactions show the HR (95% CI) and p-value for TBI x biomarker interaction term. Additive interactions show the exponentiated relative excess risk due to interaction (RERI) (95% CI) and p-values.

The analytic sample was restricted to pre-dementia plasma biomarkers measured using the Quanterix Simoa platform. TBIs that occurred one year before plasma was obtained were censored. Hazard ratios and 95% confidence intervals for incident dementia were calculated from cause-specific Cox proportional hazards regression models. Models adjusted for Visit 5 measures of age, sex, race-center, education, estimated glomerular filtration rate, and body mass index as time-invariant covariates. Multiple imputation by chained equations was employed to impute missing covariates. Inverse probability weighting was used to account for selection bias.

# **eFigure 8. Sensitivity Analysis of Covariate-Adjusted Association of Standardized Plasma Biomarkers with Incident Dementia in Late-Life by Any Traumatic Brain Injury (TBI) Status, the ARIC Study 2011-2020 (N=1,375)**

Abbreviations: Aβ, amyloid-β; CI, confidence intervals; GFAP, glial fibrillary acidic protein; HR, hazard ratio; NfL, neurofilament light chain; p-tau181, phosphorylated Tau-181; TBI, traumatic brain injury.

^Indicates that the Aβ42/Aβ40 ratio was inverted so that higher values denote greater risk of incident dementia.

^a^Biomarkers standardized to Visit 3 by mean-centring and dividing by 0.0459 for Aβ42/Aβ40 ratio, 0.7997 for log2 p-tau181, 0.6824 for log2 NfL, and 0.6885 for log2 GFAP.

^b^Biomarkers standardized to Visit 5 by mean-centring and dividing by 0.0143 for Aβ42/Aβ40 ratio, 0.6631 for log2 p-tau181, 0.6990 for log2 NfL, and 0.6951 for log2 GFAP.

^c^Change in biomarkers per decade standardized by mean-centring and dividing by 0.0270 for Aβ42/Aβ40 ratio, 0.4506 for log2 p-tau181, 0.3589 for log2 NfL, and 0.3172 for log2 GFAP.

Multiplicative interactions show the HR (95% CI) and p-value for TBI x biomarker interaction term. Additive interactions show the exponentiated relative excess risk due to interaction (RERI) (95% CI) and p-values.

The analytic sample was restricted to pre-dementia plasma biomarkers measured using the Quanterix Simoa platform. Hazard ratios and 95% confidence intervals for incident dementia were calculated from cause-specific Cox proportional hazards regression models. Models adjusted for Visit 5 measures of age, sex, race-center, education, estimated glomerular filtration rate, and body mass index as time-invariant covariates. Multiple imputation by chained equations was employed to impute missing covariates. Inverse probability weighting was used to account for selection bias.

# **eReferences**

1. Papp A, Hatzakis H, Bracey A, Wu K. ARIC hemostasis study--I. Development of a blood collection and processing system suitable for multicenter hemostatic studies. *Thromb Haemost* 1989; **61**: 15-9.
2. Rissin D, Kan C, Campbell T, et al. Single-molecule enzyme-linked immunosorbent assay detects serum proteins at subfemtomolar concentrations. *Nat Biotechnol* 2010; **28**: 595-9.
3. Wilson D, Rissin D, Kan C, et al. The Simoa HD-1 Analyzer: a novel fully automated digital immunoassay analyzer with single-molecule sensitivity and multiplexing. *J Lab Autom* 2016; **21**: 533-47.
4. Lu Y, Pike J, Chen J, et al. Changes in Alzheimer's disease blood biomarkers and associations with incident all-cause dementia. *JAMA* 2024; **332**: 1258-69
5. Inker LA, Eneanya ND, Coresh J, et al. New creatinine- and cystatin c-based equations to estimate GFR without race. *N Engl J Med 2021*; **385**: 1737-49.
6. Zhang B, Zhang C, Wang Y, et al. Effect of renal function on the diagnostic performance of plasma biomarkers for Alzheimer's disease. *Front Aging Neurosci* 2023; **15**: 1150510.
7. Janelidze S, Barthelemy NR, He Y, Bateman RJ, Hansson O. Mitigating the associations of kidney dysfunction with blood biomarkers of Alzheimer disease by using phosphorylated tau to total tau ratios. *JAMA Neurol* 2023; **80**: 516-22.
8. Van Buuren S. Multiple imputation of discrete and continuous data by fully conditional specification. *Stat Methods Med Res* 2007; **16**: 219-42.
9. Collins L, Schafer J, Kam C. A comparison of inclusive and restrictive strategies in modern missing data procedures. *Psychol Methods* 2001; **6**: 330-51.
10. Mainzer R, Nguyen C, Carlin J, Moreno-Betancur M, White I, Lee K. A comparison of strategies for selecting auxiliary variables for multiple imputation. *Biom J* 2024; **66**: e2200291.
11. von Hippel P. How many imputations do you need? A two-stage calculation using a quadratic rule. *Sociol Methods Res* 2020; **49**: 699-718
12. Rubin D. Multiple Imputation for Nonresponse in Surveys. New York, NY: Wiley, 1987.
13. Sharrett A, Patsch W, Sorlie P, Heiss G, Bond M, Davis C. Associations of lipoprotein cholesterols, apolipoproteins A-I and B, and triglycerides with carotid atherosclerosis and coronary heart disease. The Atherosclerosis Risk in Communities (ARIC) Study. *Arterioscler Thromb* 1994; **14**: 1098-1104.
14. Siedel J, Hägele E, Ziegenhorn J, Wahlefeld A. Reagent for the enzymatic determination of serum total cholesterol with improved lipolytic efficiency. *Clin Chem* 1983; **29**: 1075-80.
15. White A, Folsom A, Chambless L, et al. Community surveillance of coronary heart disease in the Atherosclerosis Risk in Communities (ARIC) Study: methods and initial two years' experience. *J Clin Epidemiol* 1996; **49**; 223-33.
16. Chambless L, Heiss G, Folsom A, et al. Association of coronary heart disease incidence with carotid arterial wall thickness and major risk factors: the Atherosclerosis Risk in Communities (ARIC) Study, 1987-1993. *Am J Epidemiol* 1997; **146**: 483-94.
17. Benton A, Hamsher K. Multilingual Aphasia Examination. Iowa City, IA: University of Iowa, 1976.
18. Wechsler D. Wechsler Memory Scale-Revised. San Antonio, TX: Psychological Corporation, 1987.
19. Knopman D, Ryberg S. A verbal memory test with high predictive accuracy for dementia of the Alzheimer type *Arch Neurol* 1989; **46**: 141-5.
20. Williams B, Mack W, Henderson V. Boston naming test in Alzheimer’s disease. *Neuropsychologia* 1989; **27**: 1073-9.
21. Reitan R. Validity of the trail making test as an indicator of organic brain damage. *Percept Mot Ski* 1958; **8**: 271-6.
22. Ryan J, Lopez S. Wechsler adult intelligence scale-III. Understanding psychological assessment. Perspectives on individual differences. New York, NY: Kluwer Academic/Plenum Publishers, 2001.
23. Gross A, Power M, Albert M, et al. Application of latent variable methods to the study of cognitive decline when tests change over time. *Epidemiology* 2015; **26**: 878-87.
24. Folstein M, Folstein S, McHugh P. "Mini-mental state". A practical method for grading the cognitive state of patients for the clinician. *J Psychiatr Res* 1975; **12**: 189-98.
25. Knopman D, Griswold M, Lirette S, et al. Vascular imaging abnormalities and cognition: mediation by cortical volume in nondemented individuals: Atherosclerosis Risk in Communities-Neurocognitive Study. *Stroke* 2015; **46**: 433-40.
26. Kurland B, Heagerty P. Directly parameterized regression conditioning on being alive: analysis of longitudinal data truncated by deaths. *Biostatistics* 2005; **6**: 2-258.
27. Kurland B, Johnson L, Egleston B, Diehr P. Longitudinal data with follow-up truncated by death: match the analysis method to research aims. *Stat Sci* 2009; **24**: 211.
28. Knopman D, Gottesman RF, Sharrett AR, et al. Mild cognitive impairment and dementia prevalence: The Atherosclerosis Risk in Communities Neurocognitive Study (ARIC-NCS). *Alzheimers Dement (Amst)* 2016; **2**: 1-11.
29. Knopman DS, Pike JR, Gottesman RF, et al. Patterns of cognitive domain abnormalities enhance discrimination of dementia risk prediction: The ARIC study. *Alzheimers Dement* 2024; **20**: 4559-4571.
